# Base editing screens map mutations affecting interferon-γ signaling in cancer

**DOI:** 10.1016/j.ccell.2022.12.009

**Published:** 2023-02-13

**Authors:** Matthew A. Coelho, Sarah Cooper, Magdalena E. Strauss, Emre Karakoc, Shriram Bhosle, Emanuel Gonçalves, Gabriele Picco, Thomas Burgold, Chiara M. Cattaneo, Vivien Veninga, Sarah Consonni, Cansu Dinçer, Sara F. Vieira, Freddy Gibson, Syd Barthorpe, Claire Hardy, Joel Rein, Mark Thomas, John Marioni, Emile E. Voest, Andrew Bassett, Mathew J. Garnett

**Affiliations:** 1 Translational Cancer Genomics, Wellcome Sanger Institute, Hinxton, UK; 2 Gene Editing and Cellular Research and Development, Wellcome Sanger Institute, Hinxton, UK; 3 Cancer, Ageing and Somatic Mutation, Wellcome Sanger Institute, Hinxton, UK; 4 Cellular Operations and Stem Cell Informatics, Wellcome Sanger Institute, Hinxton, UK; 5 Department of Immunology and Molecular Oncology, Netherlands Cancer Institute, Amsterdam, the Netherlands; 6 Oncode Institute, Utrecht, the Netherlands; 7 Instituto Superior Técnico, Universidade de Lisboa, 1049-001, and, INESC-ID, 1000-029, Lisbon, Portugal; 8 Open Targets, Cambridge, UK; 9 EMBL-European Bioinformatics Institute, Cambridge, UK

**Keywords:** base editing, cancer immunotherapy, variants of uncertain significance, functional genomics, drug resistance, cancer genetics, IFN-γ signaling, gene editing, interferon gamma

## Abstract

Interferon-γ (IFN-γ) signaling mediates host responses to infection, inflammation and anti-tumor immunity. Mutations in the IFN-γ signaling pathway cause immunological disorders, hematological malignancies, and resistance to immune checkpoint blockade (ICB) in cancer; however, the function of most clinically observed variants remains unknown. Here, we systematically investigate the genetic determinants of IFN-γ response in colorectal cancer cells using CRISPR-Cas9 screens and base editing mutagenesis. Deep mutagenesis of *JAK1* with cytidine and adenine base editors, combined with pathway-wide screens, reveal loss-of-function and gain-of-function mutations, including causal variants in hematological malignancies and mutations detected in patients refractory to ICB. We functionally validate variants of uncertain significance in primary tumor organoids, where engineering missense mutations in *JAK1* enhanced or reduced sensitivity to autologous tumor-reactive T cells. We identify more than 300 predicted missense mutations altering IFN-γ pathway activity, generating a valuable resource for interpreting gene variant function.

## Highlights

• Systematic base editing mutagenesis of the IFN-γ pathway in colorectal cancer cells
• Functional assessment of variants relevant to cancer and immune disorders
• Mutagenesis highlights functional protein domains and interaction interfaces
• Missense mutations in JAK1 alter sensitivity of tumor organoids to T cells

## Graphical abstract

**Figure:**
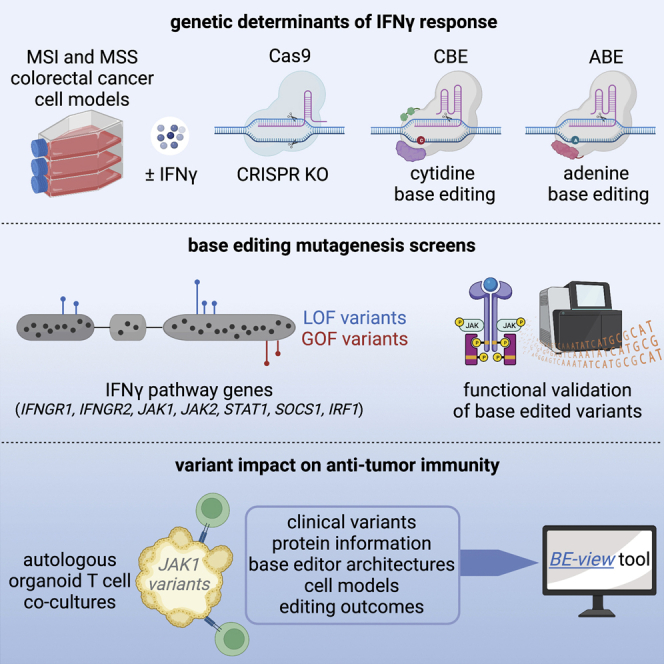

## Teaser

Coelho et al. use functional genomics to systematically analyze the genetic determinants of IFN-γ response in colorectal cancer cells. Base editing mutagenesis of key signaling components facilitates the parallel assessment of gene variant function at scale, relevant for understanding IFN-γ signaling in cancer immune surveillance and immune disorders.

## Introduction

Cellular responses to the cytokine interferon-γ (IFN-γ) are essential for normal inflammatory responses, but pathway dysfunction and disease can occur through mutation, leading to hematological malignancies and immunological disorders.^1^^,^^2^ JAK inhibitors are used to treat myeloproliferative disorders such as polycythemia vera and inflammatory disorders such as rheumatoid arthritis and ulcerative colitis,^2^ reflecting the central role of JAK-STAT signaling in these diseases. Furthermore, IFN-γ signaling in cancer cells is a critical aspect of anti-tumor immunity.^3^^,^^4^ Clinical resistance to immune checkpoint blockade (ICB), such as antibody therapies targeting programmed cell death 1 (PD-1) and CTLA-4, has been associated with somatic mutation and homozygous inactivation of IFN-γ pathway components in tumor cells,^5^^,^^6^^,^^7^^,^^8^ or inactivation of genes involved in antigen processing and presentation (e.g., *B2M*)^9^^,^^10^ that are expressed in response to IFN-γ. For example, mutations in JAK1 and JAK2 can confer resistance to ICB^5^^,^^6^ and chimeric antigen receptor T cells.^11^ Since somatic mutations in cancer are predominantly single nucleotide changes, which often result in missense mutations with unknown consequence^12^^,^^13^ (i.e., variants of uncertain significance [VUS]), interpreting their functional relevance remains challenging, representing an impediment to diagnosis, patient stratification, and the management of drug-resistant disease.

Experimental approaches are important to assess the functional effects of VUS. This is due to the ability to establish causality between VUS and disease-related phenotypes, as well as a scarcity of clinical datasets (e.g., from sequencing ICB-resistant tumors) and the infrequent occurrence of some variants in patient cohorts. One approach to prospectively assess endogenous gene variant function at scale is base editing^14^^,^^15^^,^^16^^,^^17^^,^^18^^,^^19^^,^^20^; a cluster regularly interspaced short palindromic repeats (CRISPR)-based gene editing technology that uses cytidine^21^ or adenine^22^ deaminases to install C->T or A->G transitions, respectively. Base editors achieve high editing efficiencies within the activity window, which is typically focused around positions 4–8 of the protospacer (where the PAM spans position 21–23), with minimal generation of DNA insertions and deletions.^23^

**Figure 1:**
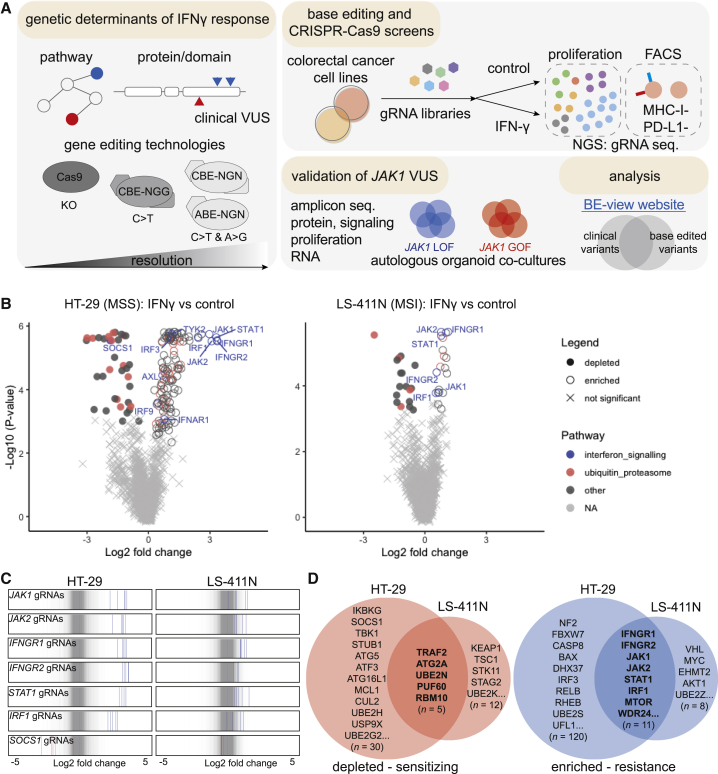
CRISPR-Cas9 screens identify mediators of IFN-γ sensitivity and resistance (A) Schematic of the integrated CRISPR-Cas9 and base editing screening approaches to identify genetic mediators of sensitivity and resistance to IFN-γ. Cas9 screens identify pathways and genes regulating IFN-γ response in colorectal cancer cell lines and base editing mutagenesis screens assess the functional consequence of VUS in key regulators. (B) Gene-level volcano plots of CRISPR-Cas9 screens comparing IFN-γ-treated and control arms. (C) gRNA-level analysis of top resistance or sensitizing genes, representing essential components of the IFN-γ pathway. (D) Common and private genes conferring sensitivity and resistance to IFN-γ in HT-29 and LS-411N CRC cell lines identified from CRISPR-Cas9 screens. Hits were selected using MAGeCK; p < 0.05 and a false discovery rate of less than 0.05. All data are the average from two independent screens performed on separate days. See also Figure S1 and Table S1.

In this study, we use CRISPR-Cas9 screening to identify mediators of sensitivity and resistance to IFN-γ in colorectal adenocarcinoma (CRC), and use cytidine base editors (CBEs) and adenine base editors (ABEs) to perform mutagenesis of the top-scoring genes, thereby systematically mapping loss-of-function (LOF) and gain-of-function (GOF) variants modulating IFN-γ pathway activity (Figure 1A), including VUS associated with diseases such as cancer.

## Results

### CRISPR-Cas9 screens identify mediators of sensitivity and resistance to IFN-γ

Mechanisms of immune evasion can be cancer-cell intrinsic,^24^^,^^25^ and thus systematically explored *in vitro* using CRISPR-Cas9 screens in cancer cell models.^26^ This approach has identified tumor IFN-γ signaling as essential for sensitivity to anti-tumor T cells, but has focused on melanoma^10^^,^^27^^,^^28^ or a limited number of mouse syngeneic cell lines,^29^^,^^30^^,^^31^^,^^32^ leaving other indications for ICB, such as CRC,^33^ relatively unexplored. To systematically evaluate genetic, cell-intrinsic determinants of IFN-γ signaling, and nominate genes for further investigation, we performed CRISPR-Cas9 screens in two *BRAF*-mutant CRC cell lines, HT-29 (microsatellite stable) and LS-411N (microsatellite unstable [MSI]) (Figure 1A). Cas9-expressing derivative cell lines^34^ were transduced with an immuno-oncology focused guide RNA (gRNA) gene knock-out (KO) library, containing 10,595 gRNAs targeting 2,089 genes with a median of five gRNAs per gene (Table S1) and selected with cytotoxic doses of IFN-γ. Screen quality was verified by efficient depletion of gRNAs targeting essential genes^35^^,^^36^ (Figure S1A) and a high correlation between independent biological screening replicates (Figure S1B).

MAGeCK^37^ (Figure 1B) and Drug-Z^38^ (Figure S1C) analyses indicated that the KO of genes involved in the regulation of IFN-γ signaling, JAK-STAT signaling, and the downstream transcriptional response, caused the strongest resistance, including *IFNGR1*, *IFNGR2*, *JAK1*, *JAK2*, *STAT1*, and *IRF1* (Figure 1B), each of which had multiple gRNAs with significant enrichment specifically in the presence of IFN-γ (Figures 1C and S1D). Changes in gRNA abundance were generally greater for HT-29, reflecting higher sensitivity to IFN-γ and a faster growth rate than LS-411N (Figure S1E). Identification of hits common to both cell lines (Figure 1D) and STRING network analysis^39^ revealed genes centered around IFN-γ signaling, protein ubiquitination, RNA processing, and mammalian target of rapamycin (mTOR) signaling (Figure S1F).

KO of *mTOR*, *AKT1*, and *WDR24* were significantly associated with resistance to IFN-γ, whereas negative regulators of *mTOR*, *TSC1*, and *STK11* were sensitizing hits, consistent with the pleiotropic, immunosuppressive effects of rapamycin, and mTOR signaling potentiating IFN-γ signaling.^40^ The inactivation of genes involved in protein degradation such as tumor suppressor genes *KEAP1* and *FBXW7* has been previously implicated in sensitivity and resistance to cancer immunotherapy, respectively.^25^^,^^32^ Interestingly, *FBXW7* was a significant resistant hit in HT-29, but not LS-411N, where *FBXW7* is already mutated.^41^ Moreover, sensitizing hits included KO of *SOCS1* and *STUB1*,^30^ which are negative regulators of IFN-γ signaling that function through inhibition and proteasomal degradation of JAK1^42^ and IFNGR1.^43^ Top-scoring regulators of apoptosis, *CASP8*, *BAX*, and *MCL1*, indicated the mode of cell death induced by IFN-γ, and support the association of *CASP8* mutations with immune evasion in TCGA pan-cancer analyses.^9^ Finally, KO of autophagy-related genes enhanced cell death in the presence of IFN-γ (Figure 1D) (*ATG2A*, *ATG5*, *ATF3*, and *ATF16L1*), consistent with autophagy mediating cancer cell resistance to anti-tumor T cells.^29^

Collectively, our CRISPR-Cas9 screens identified key nodes of resistance and sensitivity to IFN-γ in colorectal cell lines for further study, with considerable overlap with clinical reports of ICB resistance in patients^5^^,^^6^^,^^7^ and genetic screens interrogating cancer immune evasion *in vitro*^10^^,^^28^^,^^29^^,^^32^ and *in vivo*^29^^,^^30^^,^^31^ (Table S1).

### Base editing mutagenesis screening of *JAK1* with BE3-NGG

**Figure 2:**
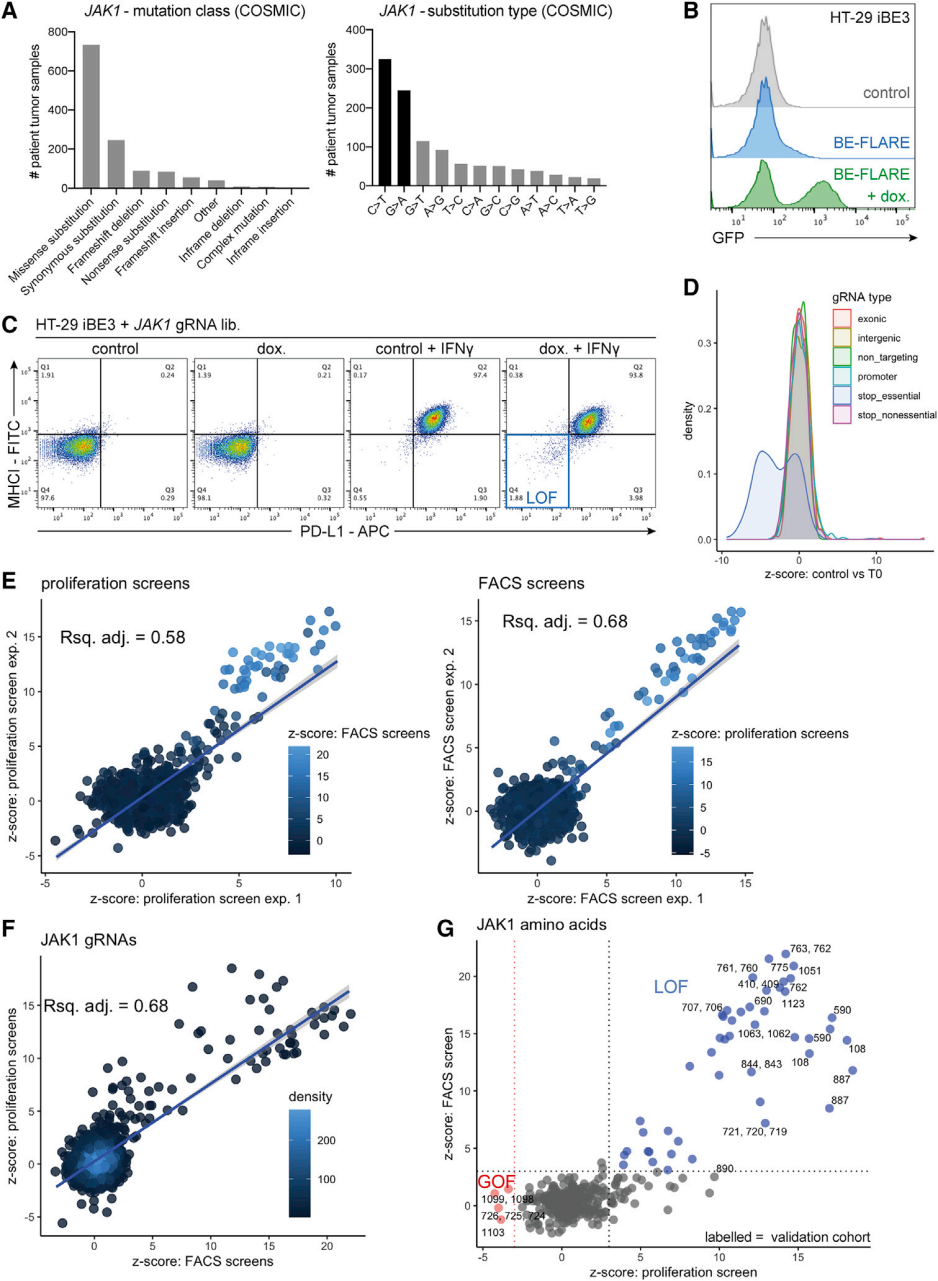
Base editing mutagenesis screening of *JAK1* variants (A) COSMIC data from patient tumor samples show *JAK1* cancer mutations are predominantly C->T and G->A missense variants. (B) BE-FLARE assessment of base editing efficiency in HT-29 iBE3 cells treated with doxycycline, based on flow cytometry analysis of a BFP (His66) to GFP (Tyr66) spectral shift. (C) FACS screening assay. After base editing of *JAK1* by the addition of doxycycline, HT-29 iBE3 cells that failed to respond to IFN-γ after 48 h were selected by FACS, as determined by the lack of induction of MHC-I and PD-L1 expression. (D) Proliferation screening assay. gRNA depletion or enrichment is indicated by z-score, comparing the control arm with the T0 (time 0) control. Base editing gRNAs designed to introduce stop codons in essential genes in HT-29 iBE3 cells are depleted. (E) Correlation between screening replicates. z-scores for gRNAs targeting *JAK1* were compared between replicates and alternative screening assays. The shaded line represents the 95% confidence interval. (F) Correlation between different base editor screening assays for JAK1 variants in HT-29 iBE3 cells. (G) Identification of LOF and GOF alleles in JAK1 protein affecting sensitivity to IFN-γ. z-scores for the base editing screens using FACS vs proliferation were plotted to select potential LOF (blue) and GOF (red) JAK1 variants. Labeling illustrates amino acid positions that were selected for further validation. All data are representative of two independent experiments or screens performed on separate days. See also Figure S2 and Table S2.

In an attempt to analyze spontaneously acquired resistance to IFN-γ, we grew HT-29 cells in the presence of IFN-γ for 2 months, but failed to derive resistant clones, necessitating the use of orthogonal approaches. *JAK1* KO caused robust resistance to IFN-γ in CRISPR-Cas9 screens, and mutation causes acquired resistance to ICB.^5^^,^^6^ *JAK1* somatic mutations in cancer are most frequently missense mutations (58.2%), with C->T or G->A transition mutations predominating (52.7%), which can be installed using CBEs (Figure 2A). Therefore, we set out to use base editing mutagenesis screens to assign functional scores to VUS in *JAK1*. To obviate potential toxicity associated with constitutive expression of deaminases, we generated doxycycline-inducible base editor 3 (iBE3)^21^ HT-29 and LS-411N cell lines. The base editing activity reporter (BE-FLARE)^44^ estimated base editing efficiencies of approximately 40% in HT-29 iBE3 (Figure 2B). Despite both cell lines having similar ploidy (approximately 3n), base editing efficiency was considerably lower in LS-411N (approximately 15%) (Figures S2A and S2B), and associated with apparent silencing of base editor expression (Figure S2C). Since LS-411N is MSI with an inactivating mutation in *MLH1*, we also tested whether mismatch repair may affect base editing by KO of *MLH1* in HT-29 iBE3 cells (Figure S2D), but found that *MLH1* was dispensable for base editing in this context (Figure S2E). We deemed high editing efficiency important because clinical resistance to ICB is associated with homozygous mutations in IFN-γ pathway components, often occurring with loss of heterozygosity.^5^^,^^6^

Using a pooled library of 2,000 gRNAs, we tiled *JAK1* in HT-29 iBE3 cells with exon-targeting gRNAs and gRNAs targeting *JAK1* promoter regions, non-targeting (NT), intergenic targeting, and control gRNAs designed to introduce stop codons in 72 essential and 28 non-essential genes (Table S2). We adopted two screening approaches: a long-term proliferation screen and a short-term flow cytometry-based assay based on major histocompatibility complex (MHC-I) and programmed death-ligand 1 (PD-L1) induction with IFN-γ (Figure 2C). gRNAs predicted to install stop codons within essential genes were significantly depleted (Figure 2D), achieving recovery of known essential genes in both screens (Figure S2F). There was no relationship between *JAK1* gRNA functional scores and the number of off-target sites (Figure S2G); however, the gRNA Rule Set 2 score^45^ (Figure S2H), or considering the immediate sequence context of the target cytidine (Figure S2I), was somewhat predictive of gRNA performance.^17^^,^^18^ Correlation between independent replicates (Figure 2E) and the proliferation and fluorescence-activated cell sorting (FACS) screens (Figure 2F), was driven by highly enriched gRNAs after positive selection with IFN-γ, representing candidate JAK1 LOF variants. As GOF variants were rare, we could only practically sort for JAK1 LOF cells by FACS, and so only recovered LOF gRNAs in the FACS screen (Figure S3A). We selected 24 gRNAs for validation studies, representing 15 LOF and 5 GOF unique variants, mostly predicted to generate missense variants with clinical precedence in cancer (Figure 2G; validation cohort). In addition, we included JAK1 Glu890 gRNA, which was unusual as it scored in the proliferation screens but not the FACS screens (Figure 2G), and the Trp690^∗^ gRNA as a control; predicted to generate a nonsense mutation observed in a CRC patient that failed to respond to ICB.^6^

### Base editing mutagenesis of the IFN-γ pathway

**Figure 3:**
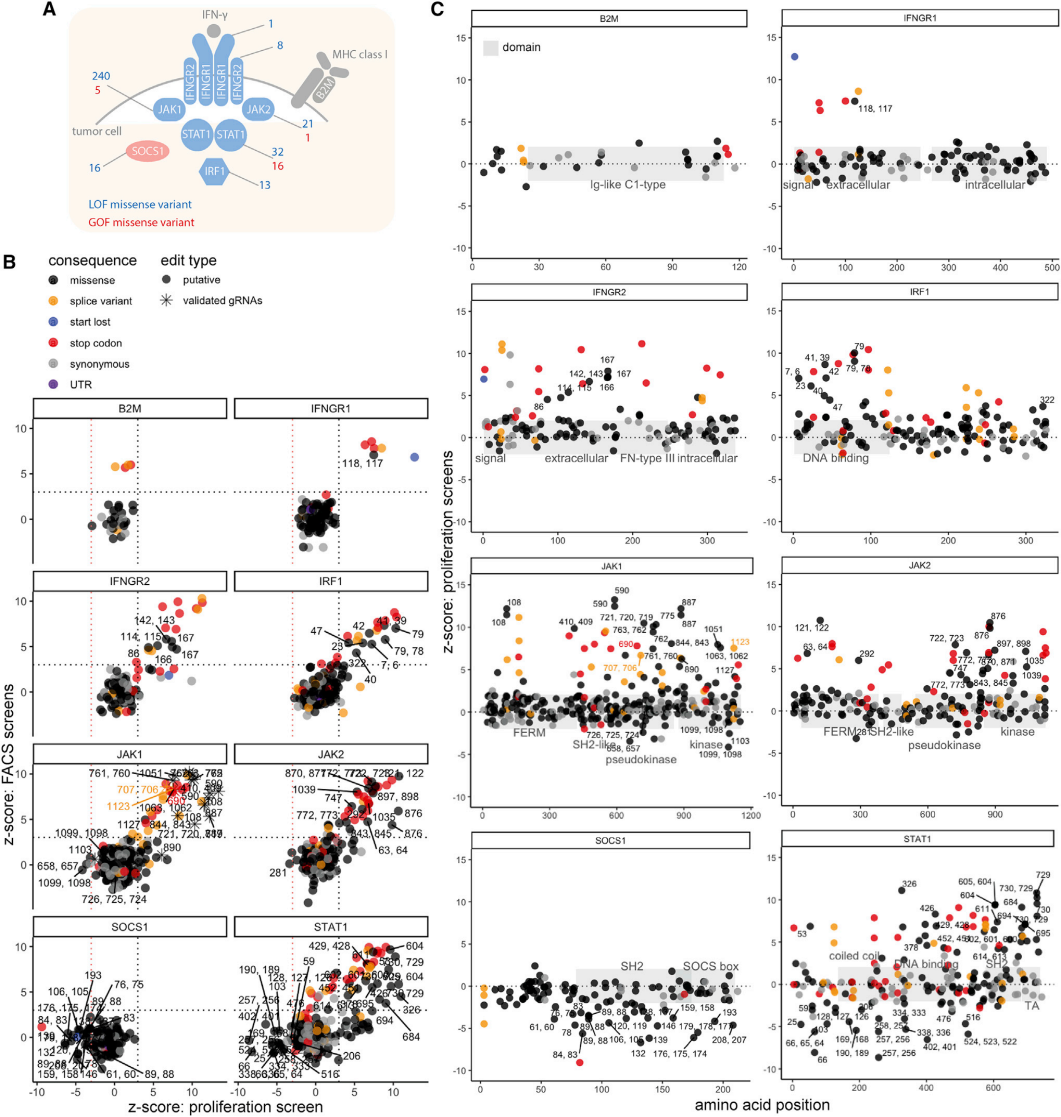
Base editing mutagenesis of the IFN-γ pathway (A) Schematic of the key mediators of IFN-γ signaling investigated in base editing screens. Depicted are top hits from our CRISPR-Cas9 screens to determine the modulators of sensitivity to IFN-γ; positive mediators are in blue and negative regulators are in red. The number of predicted LOF and GOF missense variants revealed from all base editing screens are indicated. (B) Base editor mutagenesis of core IFN-γ pathway components using HT-29 iBE3 cells reveals GOF and LOF missense mutations. The average FACS screen score is plotted against the average proliferation screen score for each gene. Positions of validated JAK1 gRNAs and amino acid positions with predicted missense LOF or GOF effect are labeled. (C) Base editing reveals the position of functional domains. Schematics of the domain architecture of proteins in the IFN-γ pathway tiled with base editing gRNAs, with the distribution of GOF and LOF amino acid positions labeled. All data are representative of two independent screens performed on separate days. See also Figure S3 and Table S2.

To achieve a more comprehensive overview of the effect of missense mutations in the IFN-γ pathway, we expanded our base editor mutagenesis screens to include top hits of our CRISPR-Cas9 screens in HT-29 iBE3-NGG cells (Figure 1B). We tiled *JAK1*, *JAK2*, *IFNGR1*, *IFNGR2*, *STAT1*, *IRF1*, *B2M*, and *SOCS1* with 4,608 gRNAs, including the previous *JAK1* gRNAs to serve as internal controls (Figure 3A and Table S2). All of these genes had 3n ploidy, except *B2M*, which was 4n.^41^ Although not a component of the IFN-γ pathway, B2M was included because of its role in MHC-I presentation and anti-tumor immunity, but it was not a hit in our initial IFN-γ survival screens as *B2M* variants should not have an effect on cell proliferation *in vitro*.

Proliferation and FACS screens were significantly correlated (Figure 3B), as were independent replicate screens (Figure S3B), each displaying a high level of enrichment of gRNAs predicted to introduce splice variants, stop codons, and start-lost mutations (Figure 3B). Once again, JAK1 Glu890 gRNA was enriched in the proliferation screen, but not in the FACS screen. Such behavior was rare for most proteins, except for the transcription factor STAT1, where a cluster of predicted LOF missense mutations was enriched only in the proliferation screen (Figure 3B), possibly indicating separation-of-function mutants. Encouragingly, we recovered validated gRNAs targeting *JAK1* in this larger screen (Table S2 and later sections). In addition to protein truncating mutations, we used *JAK1* LOF and GOF gRNAs from our validation cohort as a benchmark for setting the thresholds to call missense variants altering IFN-γ pathway activity with high confidence (Figure 3B).

Because of its short gene length and thus relatively few gRNAs predicted to install missense mutations, we only recovered highly enriched gRNAs predicted to install splice site or stop codon variants in *B2M*, and these only scored in the FACS screen, as expected. For the negative regulator of IFN-γ signaling, *SOCS1*, LOF mutations were significantly depleted. Editing of *JAK1*, *JAK2*, *IFNGR1*, *IFNGR2*, and *IRF1* predominantly gave rise to LOF missense mutations, but *STAT1* was a notable outlier; it displayed a high proportion of GOF mutations (Figure 3B). Of STAT1 LOF missense variants, 66.7% were clustered around the SH2 and transactivation domains, compared with 6.7% of nonsense and splice LOF mutations (Figure 3C). Conversely, 55.6% of STAT1 GOF mutations were within coiled-coil and DNA-binding domains, consistent with previous reports of GOF mutations within these domains in patients with chronic mucocutaneous candidiasis.^46^ LOF missense mutations in IRF1 were enriched in the DNA-binding domain (88.9%), whereas SOCS1 LOF missense variants were enriched in the SOCS box and SH2 domains (84.2%) or within the JAK inhibitory region^42^ (SOCS1 His61Tyr), demonstrating that base editing can highlight functional protein domains.

### Comparison of base editing technologies for mutagenesis screening

**Figure 4:**
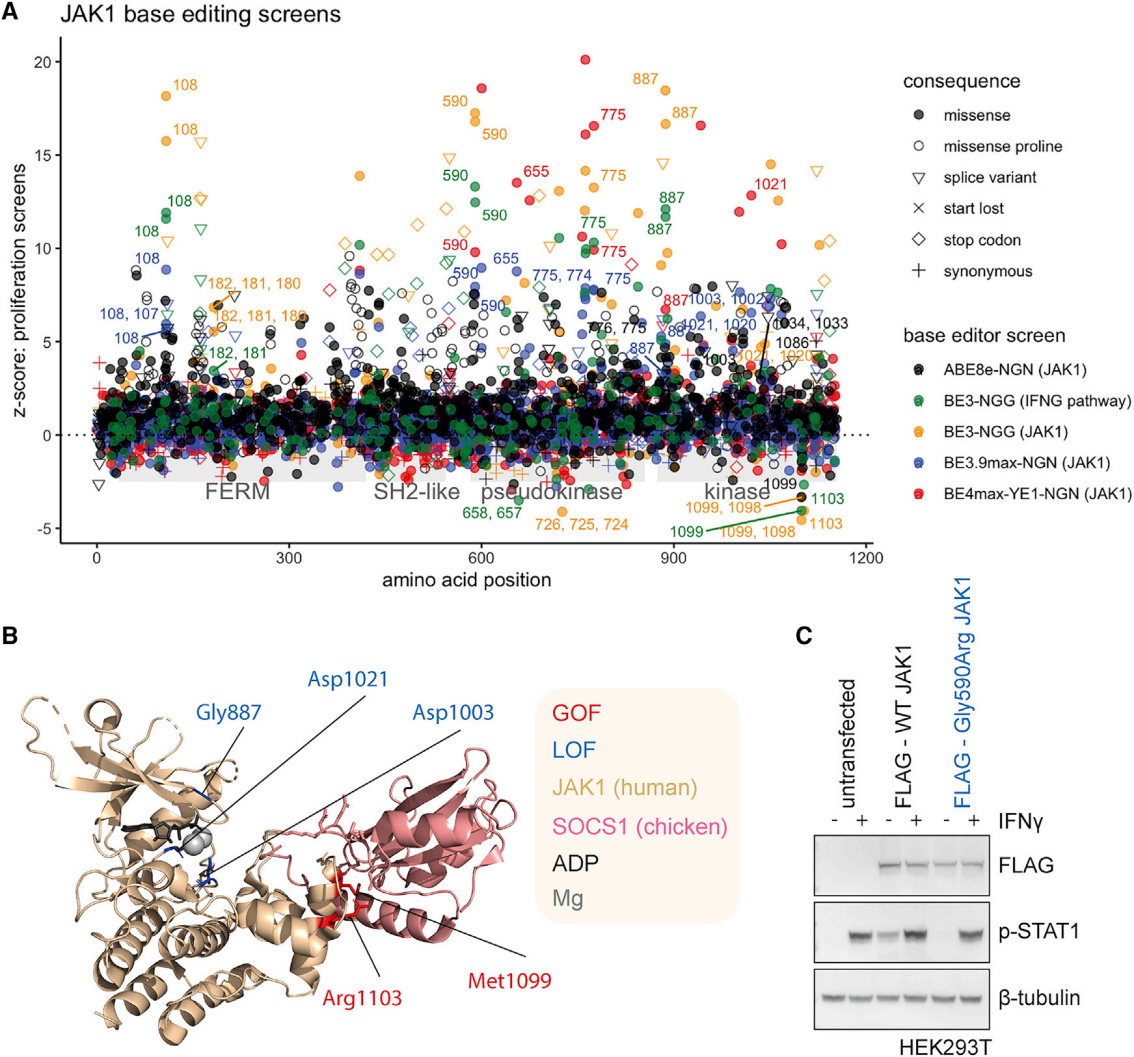
Base editing reveals JAK1 LOF and GOF variants with clinical precedence (A) Functional variant map of JAK1. z-scores from base editing proliferation screens are plotted for each gRNA across JAK1 protein domains. gRNAs installing candidate LOF and GOF positions referred to in the text are labeled with the predicted edited amino acid positions. Screen z-scores are calculated independently for each base editor and plotted together for comparison. JAK1 screening data from pathway-wide base editing screens from Figure 3 are plotted for comparison. (B) Structural insight into the mechanism of action of JAK1 LOF and GOF mutations. Crystal structure (6C7Y) shows catalytic LOF mutations (blue) proximal to the ATP/adenosine diphosphate (ADP) binding pocket in the kinase domain, and GOF mutations (red) in the binding interface with the negative regulator SOCS1. (C) Western blot of HEK293T cells overexpressing FLAG-tagged WT or Gly590Arg mutant JAK1, with or without IFN-γ stimulation for 1 h. All data are representative of two independent experiments or screens performed on separate days. See also Figures S3 and S4, Tables S2 and S3.

An analysis of amino acid mutations predicted from gRNA sequences suggested the BE3-NGG library targeted approximately 21.4% of the amino acids in JAK1. To improve the saturation of mutagenesis achievable with base editing, we used a Cas9 variant with a relaxed NGN PAM requirement,^47^ generating BE3.9max-NGN.^18^^,^^48^ Second, we sought to increase product purity by using a YE1-BE4max-NGN architecture that decreases non-C->T outcomes,^48^^,^^49^ decreases Cas9-independent off-target editing, and improves editing precision by using an engineered deaminase (YE1) with a narrower editing window.^50^^,^^51^ Finally, we used an ABE^22^ (ABE8e-NGN)^52^ to incorporate a wider variety of amino acid substitutions than can be achieved by C->T transitions alone. Using our panel of HT-29 base editor cell lines (Figure S3C), we re-screened *JAK1* with a library of 3,953 gRNAs (Table S2) targeting *JAK1* exons (Figure 4A). For NGN base editors, we detected significantly enriched gRNAs using all four PAMs (Figure S4A). ABE cannot introduce stop codons, but predicted splice variants in JAK1, which could be introduced with both CBE and ABE, were significantly enriched over NT control gRNAs in all screens (Figure S4B). Given the PAM utility and editing windows of each base editor, we predicted non-synonymous amino acid mutation coverage of JAK1 was improved to approximately 39.6% for BE4max-YE1-NGN, 50.8% for BE3.9max-NGN, 64.9% for ABE8e-NGN, and 85.1% when combining cytidine and adenine NGN mutagenesis. However, we cannot guarantee the editing efficiency of all gRNAs, so the absence of a significant score cannot be used as evidence for the lack of function of an amino acid position.

When combined, CBE and ABE editors can achieve substitutions of all 20 amino acids to at least two alternative amino acids. Substitution of amino acids with disparate chemical properties achieved larger average effect sizes, especially Leu->Pro missense mutations introduced with ABE, presumably because of the uniquely restricted φ and ψ peptide bond angles available to proline (Figures 4A and S4C). A comparison of functional scores with *in silico* predictions of variant effect (SIFT, PolyPhen, and BLOSUM62) demonstrated imperfect predictions in each case (Figure S4D), implying that high-throughput experimentation is often required to complement bioinformatic prediction of variant effect.^53^

Functional comparisons of BE3 and BE4max-YE1 editing of JAK1 confirmed the narrower editing profile of the YE1 engineered deaminase (Figure S5A), with approximately 40.5% of *JAK1* gRNAs predicted to edit only one cytosine (Table S3), but we observed a lower editing efficiency for BE4max-YE1-NGN compared with the wild-type (WT) deaminase (Figure S5B), consistent with a decreased number of significant missense, splice, and stop codon variants compared with alternative NGN base editor architectures (Figure S4B). Functional gRNAs present in both BE3 and BE4max-YE1 screens had target cytosines within the YE1 5–7 activity window (e.g., Asp775Asn gRNA 908510028), whereas out-of-window targeting gRNAs were not enriched in the BE4max-YE1 screens (e.g., Trp690^∗^ gRNA 908510274) (Figures S5A and S5C).

### Deep mutagenesis of *JAK1* reveals LOF and GOF variants with clinical precedence

To aid in the interpretation of our mutagenesis screens, we compiled a database of clinical mutations and aligned this with predicted base edited JAK1 variants. We defined clinical precedence as a non-synonymous mutation of the residue in COSMIC,^12^ TCGA, ClinVar,^54^ literature on JAK1 mutations with known effect,^1^ and data from patients receiving ICB, where cancer exome sequencing data are publicly available,^6^^,^^8^^,^^55^^,^^56^^,^^57^^,^^58^^,^^59^^,^^60^ but absence from gnomADv3.1^61^ (Table S3). LOF and GOF variants made with CBE were more likely to have clinical precedence than ABE variants, with 88% of significant CBE variants identified occurring at residues with precedence of mutation in cancer genomes, 32% of which were predicted to recapitulate the amino acid substitution with CBE (vs. 6% for ABE), perhaps reflecting the APOBEC deamination signature in cancer.^13^

Our analysis revealed candidate GOF variants in the JAK1 pseudokinase domain with clinical precedence in cancer. gRNAs targeting position Arg724 were significantly depleted with IFN-γ (Figure 4A). The predicted base edited variant, Arg724His, has been implicated in activating JAK1 signaling in acute lymphoblastic leukemia through dysregulating intramolecular inhibition of the kinase domain.^1^ Another GOF position, JAK1 Val658, is mutated in acute myeloid leukemia (AML); this residue is structurally analogous to JAK2 Val617, which is commonly mutated in polycythemia vera.^1^^,^^2^ CBE and ABE screens converged on a cluster of GOF variants in the C-terminus of the kinase domain (Met1099, Arg1103) in a known protein-protein interaction motif for SOCS1^42^ (Figure 4A), a significant negative regulator in our CRISPR-Cas9 screens. These variants presumably disrupt this interaction, increasing JAK1 protein abundance and activity (Figure 4B). Indeed, the amplification of SOCS1 has been found in patients that failed to respond to ICB,^7^ implying that this regulatory mechanism may be of clinical relevance.

LOF positions included Gly887 (Figure 4A), which is within the kinase active site, with the crystal structure,^42^ suggesting that mutation of this residue would negatively affect Mg^2+^ and adenosine triphosphate(ATP)/adenosine diphosphate coordination (Figure 4B). Other LOF mutations involving kinase catalytic residues included Asp1003 (proton acceptor), and Asp1021 (within the DFG motif), which were detected with increased (NGN) saturation (Figure 4A). ABE screens were more likely to detect sites of post-translational modification because of their ability to modify tyrosine, threonine, serine (phosphorylated), and lysine (ubiquitinated), revealing Tyr993, and the known activating Tyr1034 phosphosite as candidate LOF positions (Figure 4A) and Lys267 as a putative GOF site.

Of the candidate LOF variants found in cancer biopsies (Table S3), Gly655Asp, Gly182Glu, and Gly590Glu^56^ (Figure 4A) were all VUS detected in patients who failed to respond to ICB. In addition, JAK1 Asp775Asn has been independently verified as a LOF variant in melanoma.^6^ The overexpression of FLAG-tagged WT JAK1 in HEK293T cells resulted in a pSTAT1 signal, even in the absence of IFN-γ, and supraphysiologic stimulation with IFN-γ, whereas the JAK1 Gly590Arg mutant failed to induce STAT1 phosphorylation to the same extent in either context (Figure 4C), verifying Gly590 as a *bona fide* LOF mutation.

### Functional validation of variants conferring altered sensitivity to IFN-γ

**Figure 5:**
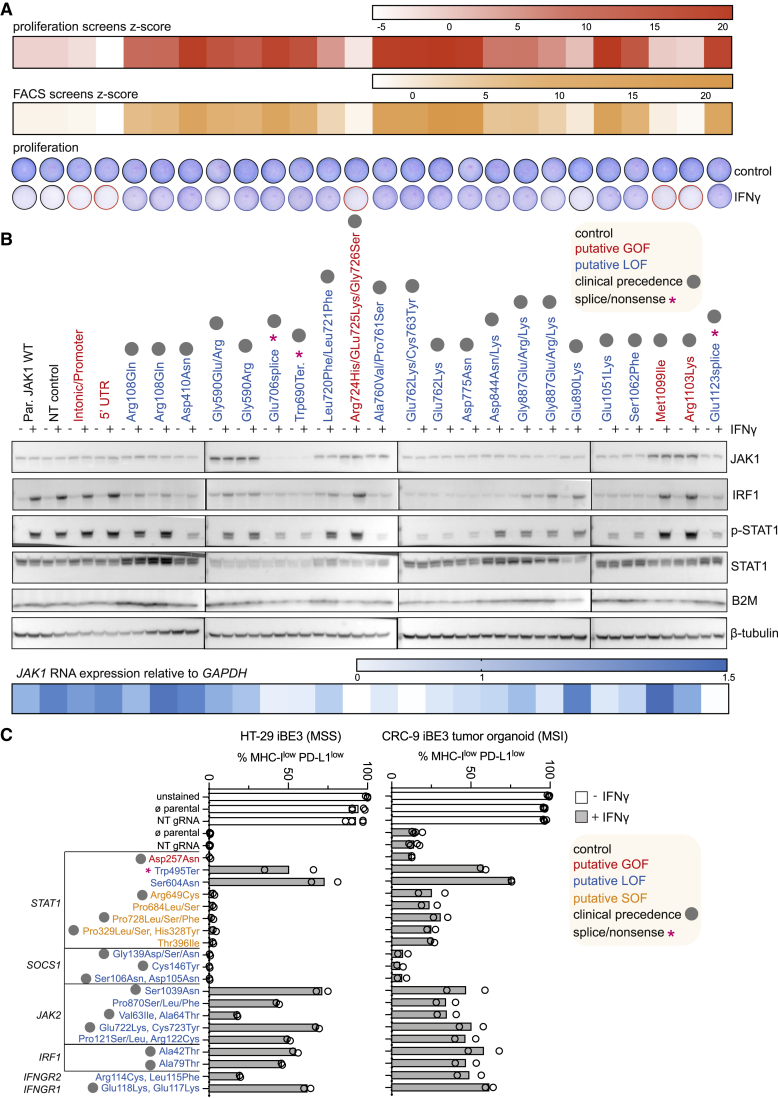
Functional validation of variants conferring altered sensitivity to IFN-γ (A) Functional validation of base editing gRNAs targeting JAK1 in HT-29 iBE3 cells. Proliferation assay: Giemsa stain following growth in the presence or absence of IFN-γ. Base editing screen z-scores for each gRNA are provided for comparison. (B) Western blot analysis of JAK1 expression and JAK-STAT signaling of corresponding JAK1 variants was performed on cells stimulated with IFN-γ for 1 h, after selection in IFN-γ for LOF variants. RNA expression, quantitative PCR analysis of *JAK1* RNA expression relative to *GAPDH* 72 h after base editing. (C) Flow cytometry analysis of MHC-I and PD-L1 expression induction following stimulation with IFN-γ for 48 h. Separation of function (SOF) variants. Bars represent the mean. All data are representative of two independent experiments performed on separate days. See also Figures S5 and S6.

We set out to functionally validate 24 gRNAs comprising our JAK1 validation cohort (Figure 2G) in an arrayed format, with multiple assays assessing cell proliferation, signaling, protein expression, RNA expression, and flow cytometry (Figures 5A–5C). This analysis was germane to screening results from multiple base editing modalities, because of their convergence on JAK1 residues within the validation cohort (e.g., Arg108, Gly590, Asp775, Gly887, and Met1099) (Figure 4A and Table S3). The growth of HT-29 iBE3 cells with engineered JAK1 variants in the presence of IFN-γ tracked with screen results, with GOF variants having no survival benefit and LOF variants having robust resistance to IFN-γ, relative to controls (Figures 5A and S5D).

Many of the candidate LOF variants had decreased levels of pSTAT1 and IRF1 induction (Figures 5B and S5D). The Met1099 and Arg1103 GOF variants had increased levels of JAK1 protein and JAK-STAT signaling, consistent with disruption of the SOCS1 binding interface and decreased E3 ubiquitin ligase-mediated destruction.^42^ Surprisingly, the Gly590 LOF variants also had increased levels of JAK1 protein, despite decreased sensitivity to IFN-γ. We speculated that increased JAK1 Gly590Arg protein could also be attributable to altered binding to SOCS1; however, we did not observe any change in binding in co-immunoprecipitation experiments (Figure S5E). JAK1 706/707 gRNA targets a splice region and had severely decreased JAK1 protein expression, similar to the clinical Trp690^∗^ nonsense control (Figure 5B). The Glu1123 splice variant decreased *JAK1* RNA abundance to levels comparable with Trp690^∗^, which we presumed was targeted for nonsense-mediated decay. However, basal *JAK1* variant RNA expression levels were generally only modestly affected, and RNA expression was not entirely indicative of JAK1 protein levels, consistent with the complex post-translational control of JAK1.^42^ Signaling assays for LOF variants were performed with pre-selection with IFN-γ to decrease contributions from remaining WT, unedited cells. However, similar results were obtained using an HT-29 iBE3 single cell clone with superior editing efficiency, which did not require prior selection with IFN-γ (Figure S5F).

Next, we generated 40 additional knock-in lines using base editing to install mutations in other IFN-γ pathway genes in HT-29, and a primary MSI colorectal cancer tumor organoid, CRC-9 (harboring *FBXW7* and *TP53* driver mutations), and used flow cytometry to assess the induction of MHC-I and PD-L1 expression upon stimulation with IFN-γ (Figure 5C). LOF mutations decreased responses to IFN-γ, except for LOF mutations in the negative regulator SOCS1, which increased the induction of MHC-I and PD-L1 (Figure S6A). Notably, we confirmed separation of function variants specific to STAT1 (Figure 3B), which had minimal effects on induction of MHC-I and PD-L1 (Figures 5C and S6A), but conferred a significant proliferation advantage in the presence of IFN-γ (Figure S6B), highlighting the value of using two screening assays and base editing to gain new, as yet poorly understood, insights into STAT1 function. In contrast, the Asp257 STAT1 GOF mutation significantly increased sensitivity to IFN-γ in both cell models (Figure S6B); this variant effect was stronger than the putative JAK1 GOF variants tested, of which only the JAK1 Met1099 variant displayed significantly increased IFN-γ sensitivity in HT-29.

### Verification of base editing genotypes with next-generation sequencing

**Figure 6:**
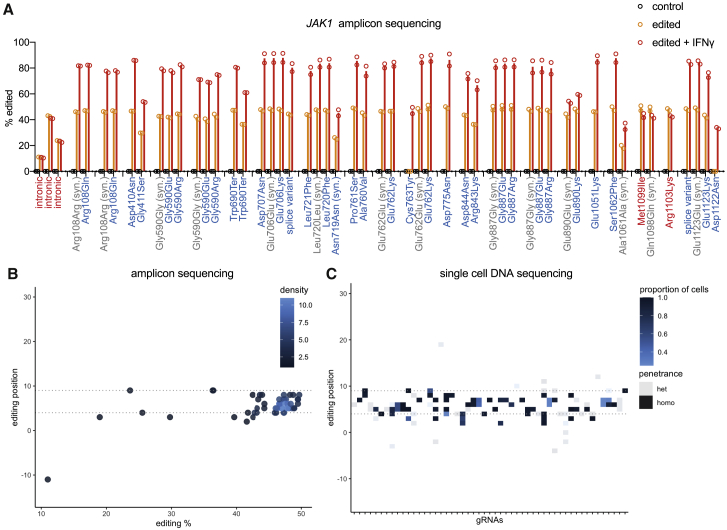
Verification of base editing genotypes with next-generation sequencing (A) Deep sequencing of *JAK1* reveals the DNA editing profile of base editor gRNAs. Editing variant allele frequency for predicted LOF and GOF gRNAs within the validation cohort measured by NGS of amplicons in control cells, base edited cells, or base edited cells with selection with IFN-γ for 6 d. Different editing outcomes are grouped by gRNA. Syn, synonymous. Data represent the mean of two independent experiments performed on separate days. (B) Amplicon sequencing assessment of HT-29 iBE3 base editing positions within the gRNA protospacers profiled in Figure 6A in the absence of IFN-γ. (C) Single-cell DNA sequencing of base editing in HT-29 iBE3 cells across 50 gRNAs reveals C->T (or G->A) editing focused in the gRNA activity window. Penetrance (zygosity) 0/1/1 is heterozygous (het), and 1/1/1 is homozygous (homo). The proportion of cells with the same gRNA assignment harboring that edit is indicated. See also Figure S6 and Table S4.

We performed amplicon sequencing of the endogenous *JAK1* loci to unambiguously assign base edited genotypes (Figure 6A). This analysis confirmed accurate predictions of base editing outcomes, detecting C->T editing focused within the BE3 activity window (approximately 4–9 relative to the PAM at position 21–23), with a minority of gRNAs (22.7%) exhibiting lower frequency edits upstream or downstream (Figures 6B and S6C). Collectively, this resulted in two unanticipated coding mutations from the validation cohort (JAK1 Asp1122Asn and Gly590Glu) caused by editing at protospacer positions 2, 3, and 11. LOF variants were enriched in the presence of IFN-γ without exception (JAK1 Glu890 was modestly enriched), verifying the associated resistance phenotype and LOF classification (Figure 6A). Co-enrichment of LOF variants with synonymous mutations (63.6% of gRNAs) implied selection for edited cells, with co-occurring neutral edits.

To more comprehensively assign the genotypes of base edits *en masse*, we used single-cell DNA-sequencing (scDNA-seq) (Figure 6C). Of the 87 gRNAs assigned *JAK1* edits, a comparison of scDNA-seq data to amplicon sequencing showed strong concordance between genotypes (Table S4). Combining amplicon and scDNA-seq datasets facilitated genotyping of edits associated with 98/665 *JAK1* targeting gRNAs. For gRNAs where we detected *JAK1* editing, predictions of amino acid changes from gRNA sequences overlapped with observed protein changes from amplicon sequencing or scDNA-seq in all cases, with 81% of predictions capturing all observed amino acid changes. An advantage of scDNA-seq over bulk amplicon sequencing is the assessment of the penetrance of editing (zygosity). Based on clinical data, we expect that homozygous editing of all *JAK1* alleles is required for a LOF phenotype,^5^^,^^6^ and this was the most frequently observed editing outcome (59% of edits). Of homozygous editing, 83% was focused within the 4–9 base editing window, compared with 76% for homozygous and heterozygous edits combined (Figure 6C). Moreover, edits outside of this activity window were private to a smaller proportion of cells with the same gRNA assignment.

For 37 gRNAs, we did not detect cells with *JAK1* edits; 4 of these gRNAs did not have target cytosines within the base editing window, 6 had exclusively GC targets in the editing window, and 2 had gRNAs with poly-T tracts that could act as U6 transcription termination signals. The remaining 25 gRNAs were expected to have a higher propensity to install base edits, giving an estimation of the false-negative rate of iBE3 cytidine base editing screens (28.7%) using our strict criteria for a calling edited genotypes (STAR Methods), although this rate varies based on the editing efficiency of each cell system. Nonetheless, the false-negative rate reinforces that we cannot interpret the lack of a significant score as evidence for a residue not being important for protein function.^18^

Taken together, these data represent a comprehensive profile of base editing outcomes at endogenous DNA loci and in single cells and indicate the predictability and precision with which variants can be installed using transient expression of base editors from a doxycycline-inducible system. For variants where the edited genotype was not assigned through sequencing, variants are predictions from gRNA sequences and based on the profiling of base editing outcomes at endogenous loci.

### Classified *JAK1* missense mutations alter sensitivity to autologous anti-tumor T cells in primary human tumor organoids

**Figure 7:**
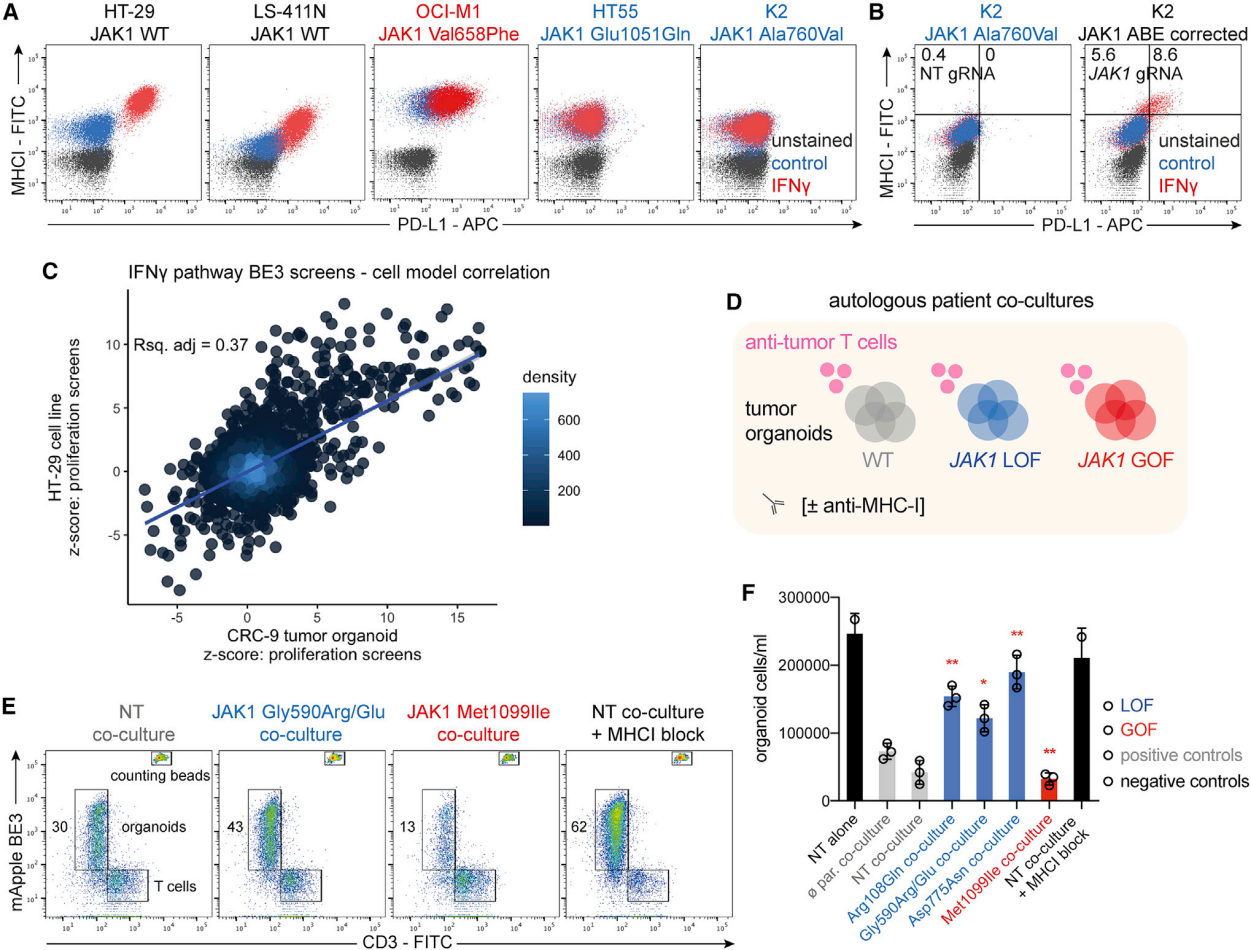
Classified JAK1 missense mutations alter tumor organoid sensitivity to autologous anti-tumor T cells (A) Flow cytometry analysis of PD-L1 and MHC-I expression in response to IFN-γ in cancer cell lines with endogenous, classified LOF or GOF mutations in JAK1. (B) Flow cytometry analysis of PD-L1 and MHC-I expression after correction of an endogenous JAK1 LOF mutation with ABE8e-NGN in K2 cells. The percentage of cells in each gate is indicated. (C) Correlation between iBE3 base editing screens in HT-29 cells and CRC-9 tumor organoids. z-scores from the IFN-γ comparison with the control arm were compared for gRNAs targeting the IFN-γ pathway. (D) Schematic of co-culture experiments to assess T cell-mediated killing of patient-derived, autologous tumor organoids (CRC-9). (E) T cell-mediated killing of autologous human tumor organoids. Flow cytometry analysis of T cells and organoids (expressing iBE3-mApple) after 72 h of co-culture. The percentage of gated organoid cells is indicated. Counting beads were used to quantify the absolute cell counts. (F) Quantification of T cell-mediated killing of autologous tumor organoids from flow cytometry analysis. Data represent the average ± standard deviation of three biological replicates and were compared against parental co-culture controls using an unpaired, two-tailed Student’s t-test (^∗∗^p < 0.01, ^∗^p < 0.05). NT, non-targeting gRNA; ø par., parental tumor organoid. All data are representative of two independent experiments performed on separate days. See also Figure S7.

To understand the broader functional implications of base editing variants, we mined an extensive collection of cancer cell models (n = 1,357) with associated exome sequencing data^41^ for pre-existing JAK1 LOF and GOF variants discovered here. The AML cell line OCI-M1 harbored the JAK1 Val658Phe GOF mutation, and 10 cell lines had homozygous inactivating frameshift or nonsense JAK1 mutations. HT55 (CRC) and K2 (melanoma) cell lines harbored homozygous Glu1051Gln and Ala760Val putative JAK1 LOF missense mutations, respectively (Figure S7A). As predicted, HT55 and K2 failed to respond to IFN-γ compared with JAK1 WT cancer cell lines, as measured by failure to induce MHC-I and PD-L1 expression (Figure 7A). OCI-M1 had relatively high basal expression of MHC-I and PD-L1, consistent with increased levels of JAK-STAT signaling, with further induction of PD-L1 upon IFN-γ stimulation. The endogenous C->T mutation in K2 cells was amenable to correction by adenine base editing. ABE8e-NGN-mediated reversion of this JAK1 mutation led to restoration of IFN-γ sensitivity (Figure 7B), verifying that this variant is responsible for resistance to IFN-γ. Interestingly, all of these cancer cell lines were derived before ICB was widely available, which suggests these variants arose from *in vivo* immunoediting^3^^,^^9^ rather than resistance to therapy.

To further assess the relevance of our findings in other cell models and in a more translational setting, we applied base editing to CRC-9 primary tumor organoids, derived from an MSI colorectal cancer patient where autologous, tumor-reactive T cells have been derived from the patient’s peripheral blood mononuclear cells (PBMCs).^62^^,^^63^ First, we reperformed the BE3-NGG base editing screen of the IFN-γ pathway components in the CRC-9 tumor organoid. There was a significant correlation between independent screening replicates (Figure S7C) and, crucially, with screening results from HT-29 iBE3 NGG (Figure 7C). These data indicate that our base editing variant data are broadly applicable and not private to a particular cell model. Furthermore, we validated clinically observed *JAK1* missense variants in CRC-9 tumor organoids using individual gRNAs, as shown by altered sensitivity to IFN-γ in three-dimensional growth assays, with LOF missense mutations at JAK1 residues 108, 590, and 775 conferring resistance (Figure S7D).

Next, we used a co-culture of matched tumor-reactive T cells with genetically engineered tumor organoids (Figure 7D) to assess T cell-mediated killing by flow cytometry (Figure 7E). After enrichment for tumor-reactive populations and expansion, co-cultured PBMCs were exclusively CD3^+^, implying a high proportion of T cells^62^^,^^63^ (Figure S7E). Strikingly, all JAK1 LOF mutant tumor organoids had significant resistance to anti-tumor T cell-mediated killing relative to WT controls, with some mutants achieving survival comparable with antibody blockade of MHC-I or growing tumor organoids in the absence of T cells (Figure 7F). Conversely, the GOF mutant Met1099Ile increased sensitivity to T cell-mediated attack. Antibody-mediated neutralization of IFN-γ in the co-culture medium significantly alleviated cytotoxicity in the WT and GOF tumor organoids, but had no effect in JAK1 LOF cells (Figures S7F and S7G), consistent with a high level of IFN-γ release from autologous anti-tumor T cells upon exposure to tumor cells,^63^ which was modestly increased with nivolumab (Figure S7H). Taken together, these data illustrate that IFN-γ pathway-variant maps from base editing screens may be predictive of anti-tumor immunity.

## Discussion

In this report, we perform 20 screens with CRISPR-Cas9 and base editors to systematically catalogue the genetic dependencies of IFN-γ response in CRC cells and map more than 300 predicted missense mutations affecting IFN-γ pathway activity. Through the use of multiple cytidine and adenine base editors, this study systematically probes protein structure and function throughout an entire signaling pathway. We provide BE-view as an online resource for exploration of these data: www.sanger.ac.uk/tool/be-view.

Tumor cell sensitivity to IFN-γ is an important determinant of ICB response in multiple tumor types.^5^^,^^6^^,^^7^^,^^8^ *JAK1* is mutated in approximately 10% of CRC and 6% of skin cutaneous melanoma, with a decrease in survival for melanoma patients with deleterious *JAK1* alterations.^6^ We detected known LOF variants (JAK1 Asp775Asn, Trp690^∗^)^6^ and assigned LOF to VUS in JAK1 that may have contributed to primary or acquired resistance to ICB resistance in the clinic (e.g., JAK1 Gly590Arg, Gly182Glu, Gly655Asp, and Pro674Ser).^56^^,^^58^ We also discovered a splice mutation in JAK1 as a LOF variant (Arg110 splice variant); however, this tumor mutation was recorded in a patient with a partial response to anti-CTLA-4.^56^ This highlights that the presence or absence of LOF variants in the IFN-γ pathway in a tumor biopsy is not an absolute determinant of ICB response^64^; rather, the outcome depends on multiple factors, including the penetrance of the mutation itself (i.e., zygosity), tumor clonal architecture, co-occurring mutations, tumor mutational burden, oncogenic signaling, tumor microenvironment, antigen presentation, and immune checkpoint engagement.^4^^,^^65^ Many of the variants we discovered with functional effects on IFN-γ signaling had clinical precedence, implying that immunoediting in cancer, particularly for immune-hot tumors, may be more prevalent than previously thought.^3^

CRISPR-Cas9 screening identified druggable targets that sensitized tumor cells to IFN-γ when inactivated, such as MCL1 and TBK1, highlighting potential ICB combination therapies in CRC. In line with this, TBK1 inhibition has been reported to increase immune reactivity to tumor organoids *ex vivo*.^66^ Interestingly, inactivation of *KEAP1*, *FBXW7*, *NF2*, and *STK11*, modulated sensitivity to IFN-γ, emphasizing important non-cell autonomous roles for these tumor suppressor genes. Although we found KO of *STK11* sensitized to IFN-γ, *STK11* mutation is associated with resistance to immunotherapy in lung adenocarcinoma,^67^ implying potential tissue-type or genotype-specific differences, and highlighting that our reductionist *in vitro* approach does not consider the potential effects on immune cells. KO of *NF2* resulted in an increased resistance to IFN-γ and has also been linked to BRAF inhibitor resistance,^45^^,^^68^ consistent with an overlap between ICB resistance and mitogen-activated protein kinase inhibitor resistance pathways,^69^ with possible implications for the efficacy of ICB in melanoma patients pre-treated with BRAF inhibitors.

IFN-γ signaling through the JAK-STAT pathway is not only relevant for cancer immunotherapy, but also underpins pathology in myeloproliferative neoplasms, chronic mucocutaneous candidiasis, primary immunodeficiency, and several inflammatory diseases.^1^^,^^2^ The molecular understanding of JAK-STAT signaling has been hindered by the lack of a full-length crystal structure of JAK1 and the complex intra-molecular regulation by the JAK1 pseudokinase domain.^1^ We report base editing screens mapping LOF and GOF variants in key regulatory regions across JAK1, including catalytic residues (ATP coordination), post-translational modifications, the pseudokinase-kinase domain interface, and inter-molecular protein-protein interactions with SOCS1, demonstrating that base editing may be used to understand complex protein biology without prior detailed structural information.^70^

Our study provides a resource for improving the interpretation of IFN-γ pathway variants in diseases such as cancer, and highlights the potential of semi-saturating base editing mutagenesis, which we envisage will complement SGE,^71^ *in silico*,^72^ and prime editing^73^ approaches in establishing the functional consequence of genetic variation.

## STAR★Methods

### Key resources table

**Table:** 

REAGENT or RESOURCE	SOURCE	IDENTIFIER
**Antibodies**		

PD-L1	Thermo Fisher Scientific	Cat.#17-5983-42
MHC-1	Biolegend	Cat.#311404
MHC-1 blocking	Thermo Fisher Scientific	Cat.#MA1-19027
PD-1 blocking	Selleckchem	Cat.#A2002
STAT1	Cell Signaling Technology	Cat.#9172S
JAK1	Cell Signaling Technology	Cat.#50996
pSTAT1	Cell Signaling Technology	Cat.#9167
IRF1	Cell Signaling Technology	Cat.#8478
β-tubulin	Cell Signaling Technology	Cat.#2146
β-actin	Cell Signaling Technology	Cat.#3700
FLAG	Sigma-Aldrich	Cat.#F3165
CD3	Thermo Fisher Scientific	Cat.#11-0038-42
IFNγ blocking	Thermo Fisher Scientific	Cat.#16-7318-81
CD28	Thermo Fisher Scientific	Cat.#16-0289-81
B2M	Cell Signaling Technology	Cat.#12851
MLH1	Cell Signaling Technology	Cat.#3515
HA	Cell Signaling Technology	Cat.#3724

**Bacterial and virus strains**		

DH5-α *E*. *coli*	NEB	Cat.#C2987I
ElectroMAX Stbl4 *E*. *coli*	Thermo Fisher Scientific	Cat.#11635018

**Biological samples**		

CRC-9 tumor organoid and autologous PBMC cultures	Cattaneo et al, 2019^62^	N/A

**Chemicals, peptides, and recombinant proteins**		

IFNγ	Thermo Fisher Scientific	Cat.#PHC4031
FuGENE HD	Promega	Cat.#E2311
IL-2	Thermo Fisher Scientific	Cat.#CTP0021
BME	R&D Systems	Cat.#3433-010-R1

**Critical commercial assays**		

IFNγ ELISA	Thermo Fisher Scientific	Cat.#EHIFNG
CellTiter-Glo	Promega	Cat.#G7570
Mission Bio Tapestri single cell sequencing	Mission Bio	N/A

**Deposited data**		

NG BE screens 1 and 2	This paper	ERP131485
NGG IFNG8 BE screens 1 and 2	This paper	ERP130865
amplicon seq. of JAK1	This paper	ERP131486
NGN ABE and CBE screens 1 and 2	This paper	ERP136370
JAK1 BE NGG screen 2	This paper	ERP131487
JAK1 BE NGG screen 1	This paper	ERP136387
CRISPR IO KO screen 2	This paper	ERP136388
CRISPR IO KO screen 1	This paper	ERP136389
CRC-9 NGG IFNG8 BE screens 1 and 2	This paper	ERP137489
MissionBio single cell DNA seq.	This paper	ERP133355

**Experimental models: Cell lines**		

HT-29	NCI	RRID: CVCL_0320
LS-411N	ATCC	RRID: CVCL_1385
OCI-M1	DSMZ	RRID: CVCL_2149
HT55	ECACC	RRID: CVCL_1294
K2	Massachusetts General Hospital, Hensin Tsao	RRID: CVCL_AT85
HEK293T	ATCC	RRID: CVCL_0063

**Oligonucleotides**		

Primers are listed in Table S7	This paper	N/A

**Recombinant DNA**		

BE-FLARE gblock from IDT	IDT and Coelho et al. 2018^44^	N/A
CLYBL-hNGN2-BSD-mApple	Michael Ward lab	Addgene plasmid #124229
YE1-BE4max-Cas9NGN	Doman et al. 2020^50^	Addgene plasmid #138159
Mammalian expression plasmid for transfection	Ran et al. 2013^85^	Addgene plasmid #48140
pKLV2-BFP-Puro lentiviral hU6 gRNA expression vector	Tzelepis et al. 2016^86^	Addgene plasmid #67974
iBE3 CLYBL	This paper	Addgene plasmid #174569
iBE4max YE1 NG CLYBL	This paper	Addgene plasmid #174570
HA-SOCS1	This paper	Addgene plasmid #174571
FLAG-JAK1	This paper	Addgene plasmid #174572
FLAG-JAK1Gly590Arg	This paper	Addgene plasmid #174573

**Software and algorithms**		

Prism 8	GraphPad	N/A
R	Comprehensive R Archive Network R project	N/A
FlowJo	Tree Star	N/A
PyMOL 2.4.1	PyMOL	N/A

**Other**		

www.sanger.ac.uk/tool/be-view	This paper	N/A

### Resource availability

#### Lead contact

Requests for further information and reagents should be directed to the lead contact, Mathew Garnett (mathew.garnett@sanger.ac.uk).

#### Materials availability

Plasmids from this article will be available from Addgene following publication (Table S5).

### Experimental model and subject details

#### Cell lines

All cell lines were mycoplasma tested and verified by STR profiling. Cells were maintained in a 5% CO_2_, 95% air, humidified incubator at 37°C, in RPMI supplemented with 1X GlutaMAX, 1X penicillin-streptomycin and 10% FCS (Thermo Fisher Scientific). Human cancer cell lines used in this study (HT-29, LS-411N, OCI-M1, HT55, K2 and HEK293T), RRID identifiers, and their source, are listed in the key resources table.

#### Primary cell cultures

PBMCs and CRC-9 tumor organoids were from the Netherlands Cancer Institute (NKI). CRC-9 is genetically female. Derivation of tumor organoids, enrichment of tumor reactive T cell populations from patient PBMCs were performed as described.^62^ Cells were maintained in a 5% CO_2_, 95% air, humidified incubator at 37°C.

### Method details

#### Cell culture

Where indicated, CellTiter-Glo proliferation assays (Promega) were performed to assess drug response following manufacturer’s instructions. For the long-term culture of HT-29 in IFNγ to derive resistant cells, we treated cells with a pre-optimized dose that killed ∼80% of parental cells (1,000 U/mL; Thermo Fisher Scientific), and refreshed the media with the addition of IFNγ twice a week for two months.

#### Tumor organoid culture

Growth and maintenance of CRC-9 tumor organoids in 3D was achieved by growth in 80% basement membrane extract (BME; R&D Systems). Co-culture killing assays were performed as described.^62^ Briefly, PBMCs were cultured in anti-CD28 coated plates for 24 h with IL-2 (150 U/mL; Thermo Fisher Scientific). CRC-9 cells were pre-stimulated with IFNγ (Thermo Fisher Scientific, 400 U/mL) overnight to increase MHC-I expression, then seeded in suspension in non-tissue culture treated 96 well plates at a 3:1 E:T ratio for 72 h, with or without anti-CD28 coating, nivolumab (20 μg/mL; Selleckchem), MHC-I blocking antibody (W6/32; 50 μg/mL) and IFNγ neutralizing antibody (NIB42; 60 μg/mL) in RPMI supplemented with human serum and primocin (Invivogen). T cell mediated killing of CRC-9 tumor organoids is dependent on MHC-I, pre-exposure of organoids to IFNγ to increase MHC-I expression and antigen presentation, but not PD-1 inhibition with nivolumab, or CD28 co-stimulation (Figure S8). Cells were harvested and stained with anti-CD3 FITC antibody (UCHT1; Thermo Fisher Scientific, 1:100), washed in FACS buffer before the addition of DAPI and flow cytometry analysis. 123count eBead counting beads (Thermo Fisher Scientific) allowed for quantification of absolute cell counts based on volumetric measurements from bead counts. The IFNγ ELISA assay was performed on neat cell culture medium from the co-culture according to manufacturer’s instructions (Thermo Fisher Scientific; #EHIFNG). Base editing screens with tumor organoid CRC-9 was performed in suspension.

#### Molecular biology and cloning

BE-FLARE reporter was synthesized as a gblock (IDT), essentially as described^44^ except where His66 codon was changed from CAC to CAT such that a single base edit can convert BFP to GFP. The gblock was integrated into a KpnI-EcoRI digested pKLV2-gRNA expression lentiviral plasmid by Gibson assembly (NEB), expressing a BE-FLARE gRNA (5′- GCTCATGGGGTGCAGTGCTT-3′).

For generation of doxycycline-inducible base editing plasmids, we digested CLYBL-hNGN2-BSD-mApple^74^ with BamHI and PmeI (thus removing hNGN2; Addgene plasmid #124229) as a backbone and used Gibson assembly to insert PCR derived fragments containing BE3,^44^ YE1-BE4max-Cas9NGN (Addgene plasmid #138159), BE3.9max-Cas9NGN,^18^^,^^48^ or ABE8e-Cas9NGN.^52^

To generate N-terminally-tagged, human, HA-SOCS1 and FLAG-JAK1 or FLAG-JAK1Gly590Arg mutant constructs, we used Addgene plasmid #48140 as a transient expression vector backbone by removing Cas9 and GFP with EcoRI-AgeI digestion (NEB), and inserting three overlapping gBlock dsDNA fragments for *JAK1* cDNA, or one gBlock for *SOCS1* cDNA (IDT) by Gibson assembly (NEB). All plasmid inserts were fully sequence verified by Sanger sequencing (Eurofins).

#### Base editor cell line generation

We knocked in base editing machinery by co-transfecting (FuGENE HD; Promega) with a plasmid encoding Cas9 and a gRNA targeting the human CLYBL locus (5′-ATGTTGGAAGGATGAGGAAA-3′), and a plasmid encoding the tet-ON base editor, blasticidin resistance and mApple expression cassettes within CLYBL homology arms. HR rates were increased by overnight pre-incubation of the cells with DNA-PK inhibitor (1 μM AZD7648). We selected transfected cells in blasticidin (10 μg/mL; Thermo Fisher Scientific) for four days and then maintained cells in 5 μg/mL thereafter. Pools were further selected by FACS for mApple expression (all positive cells). Base editing efficiency was tested using BE-FLARE.^44^ For BE3.9max NGN and ABE8e NGN, clonal lines were used for screening, which were assessed for editing activity using BE-FLARE^44^ (CBE) or a stop codon GFP reporter^75^ (ABE).

*MLH1* KO cell line clones were generated by transient transfection of a Cas9-T2A-EGFP expression plasmid (Addgene Plasmid #48140), with co-expression of an *MLH1*-targeting gRNA (5′-GCACATCGAGAGCAAGCTCC-3′), which was introduced by Golden Gate into the BbsI site of the same plasmid. Single transfected cells were selected by EGFP expression by FACS into 96 well plates for screening by PCR and Western blotting.

#### Library production

gRNAs were designed using the Wellcome Sanger Institute Genome Editing (WGE) tool^76^ https://wge.stemcell.sanger.ac.uk. We extracted all NGG or NGN gRNAs that had only one perfect genomic match, and no additional genomic matches with a single nucleotide mismatch. For JAK1 NGG screens, this generated a total of 665 exonic NGG guides that overlapped with *JAK1* on both strands, including gRNAs with editing windows flanking *JAK1* exons to capture variants affecting splicing. In addition, we included 391 NGG gRNAs overlapping *JAK1* promoter regions (GRCh38 1:64,964,978–1:64,967,543). Stop-essential base editing gRNA controls were selected from the iSTOP database.^77^ ssDNA oligonucleotide libraries (Twist Biosciences) were resuspended and PCR amplified (KAPA HiFi HotStart ReadyMix; Roche) for 10 cycles with the addition of Gibson homology arms in the primer sequences. After PCR purification (AMPure XP SPRI beads; Beckman Coulter), we performed Gibson assembly (NEB) reactions at a 5:1 insert to vector ratio, with a BbsI-digested pKLV2-BFP-Puro lentiviral hU6 gRNA expression vector as the recipient vector^34^^,^^78^ (Addgene plasmid #67974). After ethanol precipitation, we performed multiple electroporations (ElectroMAX Stbl4 cells; Thermo Fisher Scientific) to maintain library complexity. Transformation efficiency was verified by serial dilution of the liquid culture onto LB + Amp agar plates. Library plasmid pools were propagated in liquid culture in LB with ampicillin (100 μg/mL) at 30°C overnight and extracted (Qiagen).

HEK293T cells were co-transfected with psPAX2, pMD2.G and the lentiviral gRNA plasmid at a 3:1:5 mass ratio using FuGENE HD (Promega) in Opti-MEM (Thermo Fisher Scientific). Media was refreshed the next day and viral supernatant was harvested 72 h post-transfection, filtered and frozen. Thawed viral supernatant titer was assessed by infection of HT-29 cells, always in the presence of 8 μg/mL polybrene (Sigma-Aldrich), and 48 h later, measuring BFP expression by flow cytometry.

#### CRISPR-Cas9 KO screens

A custom gRNA library was manually designed from an extensive literature search, generated (Oxford Genetics), titrated using an mCherry fluorophore, and used at a viral titer that achieved 30–50% infection in HT-29 and LS-411N cells stably expressing Cas9.^34^ Cells were selected with puromycin for 4 d (2 μg/mL and 1 μg/mL, respectively), maintaining 300 X coverage, with a time 0 (T0) control sample taken 7 d after infection. 10 d after infection, cells were selected with IFNγ (2000 U/mL; Thermo Fisher Scientific) for a total of 7 d with the IFNγ arm having IFNγ media refreshed after 4 d and the control arm being passaged after 4 d. Each screen was performed independently twice on separate days.

#### Base editing screens

Base editing screens were performed with a gRNA coverage of 400-1000-fold. We adopted viral doses achieving 30–50% infected cells. For proliferation screens, as with the CRISPR-Cas9 KO screens described above, we selected cells for 4 d with puromycin, a T0 sample was taken at 6 d post-infection, then doxycycline (1 μg/mL) was added for 3 d to induce base editing, followed by selection with IFNγ (2000 U/mL; Thermo Fisher Scientific) for 7 d. For the FACS screens, the library-transduced and puromycin-selected cell population was base edited by the addition of doxycycline 10 d after infection for 3 d, and 14 d after infection, IFNγ (400 U/mL) was added to induce PD-L1 and MHC-I expression for 48 h before FACS. Due to lower overall editing efficiencies for BE4max-YE1 compared to BE3, we extended the selection with IFNγ from 7 days to 14 days and did not perform a FACS selection assay to maintain good library representation. All screens were performed independently twice on separate days.

#### FACS and flow cytometry

Cells were harvested, washed once in FACS buffer (0.5% FCS, 2 mM EDTA in PBS) before staining on ice for 30 min in the dark with anti-PD-L1 (MIH1; APC) and anti-MHC-I (W6/32; FITC; both 1:100 dilution; Thermo Fisher Scientific), and washed twice in FACS buffer and adding DAPI (1 μg/mL; Thermo Fisher Scientific) before analysis (LSRFortessa; BD Biosciences). For base editing screens, FACS was used to sort approximately 250,000 LOF cells (BD Influx cell sorter; BD Biosciences), which were expanded for seven days in the absence of IFNγ before DNA extraction. For experiments with HT55 and K2, cells were treated with IFNγ (400 U/mL; Thermo Fisher Scientific) for 48 h before analysis. FACS data were analyzed using FlowJo software. For *JAK1* variant SNP correction in K2 cells, we generated ABE8e-NGN doxycycline-inducible derivative and introduced the lentiviral gRNAs 5′-GAGGAACAATCCATGGGATT-3′ (JAK1) or 5′-GCTGATATATACGACAAGCC-3′ (NT control), as described above. Three days after addition of doxycycline (1 μg/mL), we stimulated the cells with IFNγ (400 U/mL; Thermo Fisher Scientific) for 48 h before flow cytometry analysis.

#### Next-generation sequencing

Amplicon sequencing was performed as described^79^ with primers listed in Table S7. Amplicons for *JAK1* 5′UTR and 724 positions failed quality control. For gRNA sequencing, genomic DNA was extracted from cell pellets from CRISPR or base editing screens (DNeasy Blood & Tissue; Qiagen), gRNA DNA sequences were PCR amplified (empirically determined number of cycles; KAPA HiFi HotStart ReadyMix; Roche), SPRI purified (AMPure XP SPRI beads; Beckman Coulter) and quantified (Qubit; Thermo Fisher Scientific). In addition, plasmid DNA from the original library always served as a control in screening experiments. PCR products were then indexed with a second round of PCR (8–10 cycles) with unique identifier sequences and Illumina adapters, SPRI-purified, quantified (Bioanalyzer; Agilent), pooled in an equimolar ratio, quantified by qPCR and sequenced on a HiSeq2500 (Illumina) with a custom sequencing primer (5′-TCTTCCGATCTCTTGTGGAAAGGACGAAACACCG-3′) for 19 bp single-end reads of the gRNA on Rapid Run Mode.

#### Validation experiments

Individual gRNAs were cloned in an arrayed format using a Golden Gate-based approach. We designed primers encoding a gRNA with BbsI overhangs and an additional G for hU6 RNApolIII transcription (Forward: 5′-CACC**G**NNNNNNNNNNNNNNNNNNN-3′ and Reverse: 5′-AAACNNNNNNNNNNNNNNNNNN**C**-3′), annealed by boiling and slowly cooling to room temperature, and then ligated duplexes with a BbsI entry vector, BbsI-HF (NEB), T4 DNA ligase and buffer (NEB), 1X BSA (NEB) for 30X cutting (37°C) and ligating (16°C) cycles, before heat-shock transformation of DH5-α *E*. *coli* (NEB). All gRNA sequences used can be found online through the BE-view app.

#### Western blotting

Cells were lysed with 4X sample loading buffer (8% SDS, 20% β-mercaptoethanol, 40% glycerol, 0.01% bromophenol blue, 0.2 M Tris-HCL pH 6.8) supplemented with benzonase (Sigma) to digest genomic DNA. Samples were boiled for 5 min at 95°C before SDS PAGE (Thermo Fisher Scientific). PVDF membranes were probed with the following primary antibodies: STAT1 (#9172S), JAK1 (#50996), p-STAT1 (#9167), β-tubulin (#2146), IRF1 (#8478), β-actin (#3700), B2M (#12851), MLH1 (#3515) (Cell Signaling Technology). Secondary antibodies were conjugated to horseradish peroxidase.

For validation experiments, LOF mutant JAK1 edited cells were pre-selected with IFNγ for 5 days prior to re-stimulation to enrich for edited cells. These experiments were performed without pre-selection with similar results but smaller differences due to the presence of unedited cells. For stimulation of JAK-STAT signaling, cells were treated with IFNγ (400 U/mL; Thermo Fisher Scientific) for 1 h.

#### Immunoprecipitation

HEK293T cells were transfected with FLAG-JAK1 or FLAG-JAK1Gly590Arg and HA-SOCS1 (FuGENE HD, Promega). 72 h later, cells were stimulated with IFNγ (400 U/mL; Thermo Fisher Scientific), or RPMI complete medium as a control, for 1 h before lysis with lysis buffer (20 mM Tris-HCl pH 7.4, 137.5 mM NaCl, 10% glycerol, 1% Triton X-100) supplemented with benzonase and protease-phosphatase inhibitor cocktail (Sigma-Aldrich). 25 μl of protein G Dynabeads (Thermo Fisher Scientific) were conjugated to 1 μg of anti-FLAG antibody (M2; Sigma-Aldrich) for each immunoprecipitation, which was carried out overnight at 4°C with inversion. The following day, beads were washed with wash buffer (lysis buffer with 0.1% Triton X-100), before elution with 4X sample loading buffer and SDS-PAGE. We used beads alone (without anti-FLAG antibody) as a control for binding specificity.

#### Mission Bio single-cell DNA sequencing

HT-29 iBE3 cells were single cell cloned to derive a highly efficient editing line, as verified by BE-FLARE. Edited HT-29 cells containing gRNAs were dissociated using TrypLE (Thermo Fisher Scientific) and filtered through a 30 μm filter to produce a single cell suspension (4,000 cells per μL) and loaded onto the Mission Bio Tapestri machine for single-cell amplicon genotyping. We genotyped 4,302 cells and assigned endogenous genotypes by sequencing across 40 amplicons, targeting *JAK1* exons and the gRNA expression cassette. A custom primer panel was designed by Mission Bio to amplify JAK1 exons and promoter region, as well as the gRNA sequence within a single cell. Primers and custom thermal cycling parameters used for PCR are listed in Table S7. The Tapestri DNA Pipeline On-prem was used for QC, alignment and cell calling. For each cell, variant calling was performed using GATK HaplotypeCaller. gRNA UMIs were introduced into the JAK1 NGG library using an iBAR hexanucleotide barcoding approach.^80^ A probabilistic mixture model of skewed t-distributions was used to assign gRNAs to cells based on gRNA UMI counts normalized by the overall UMI counts for the cells across all amplicons. According to this probabilistic model, cells were included with at least a 99% probability of having at least one gRNA, and a probability of at most 1% of having multiple gRNAs. Only 1/39 (2.6%) control gRNAs not targeting *JAK1* were assigned a *JAK1* edit (Table S4), indicating accurate cell gRNA assignment. Three edits at 6767, 8567, 8563 nucleotides away from the gRNA start position corresponding to two gRNAs are not shown in Figure 6, and are likely associated with rare gRNA misassignment. For the HT-29 iBE3 cell clone, scDNA-seq also identified two intronic *JAK1* C->T SNPs (1: 64965916, rs12127284, and 1: 64860287, rs310224) and one heterozygous, synonymous C->T *JAK1* SNP (1: 64837976; rs12129819), in a large proportion of cells, and so these were not deemed gRNA-dependent editing events in downstream analyses. gRNAs with at least three cells uniquely assigned are plotted, where the edit is present in at least three cells equating to at least 25% of the cells with that gRNA. A variant call for two or three mutant alleles (>60% allele frequency) was deemed high-confidence and are shown in Figure 6 for the triploid cell line HT-29.

#### Data analysis

To call SNPs from amplicon sequencing, we used CaVEMan^81^ and BCFtools.^82^ Variant allele frequency (VAF) was calculated using vafCorrect,^83^ and variants with <1% VAF were filtered out. For COSMIC analysis, mutations and frequencies were downloaded from https://cancer.sanger.ac.uk/cosmic in January 2021. For visualization of crystal structures, we used PyMOL (version 2.4.1), for graphs we used GraphPad Prism (version 8) or R ggplot2 (3.3.0). For CRISPR-Cas9 and base editing screens, we filtered out any gRNAs with 0 read counts in the control samples. Log2 fold-changes (L2FC) were calculated from normalized read counts (normalized reads per million = gRNA reads/total reads for the sample x 1,000,000 + 1 pseudocount). For CRISPR-Cas9 screens, MAGeCK analysis was performed using default parameters, except that normalization is set to ‘none’, as the input corrected counts had already been normalized. A false discovery rate cut-off of 5% (FDR ≤0.05) and significance p < 0.05 was applied to identify the candidate genes for follow-up studies. For base editing screens, we implemented DrugZ to calculate a gene level z-score for each fold change using an empirical Bayes estimate of the standard deviation. We calculated z-scores using normalization by L2FC from nonessential/intergenic/non-targeting control gRNAs. Analyses with L2FC and z-scores gave similar results. For base editing screens, we considered the base edits from each gRNA as single mutations or the mutation of all cytosines or adenines in the base editing window and used VEP^84^ to assign amino acid changes. For BE3 and ABE8e we assumed a lenient window of 4–9 and for BE4max-YE1 NGN we used a window of 5–7, where 20–23 is the PAM. We focused our analysis on VEP output of MAINE selected canonical protein coding transcripts. For annotation of edit consequence, we consolidated multiple predicted consequences by giving priority to the most deleterious as follows: stop gain > start loss > splice variant > missense > UTR > synonymous variant. For base editing screens, we filtered out samples with <100 gRNA read counts for any sample in either replicate, and one gRNA that was over-represented (>50,000 reads) in the library. For CRC-9 tumor organoid screens, we also excluded seven gRNAs from downstream analysis that had > 3-fold read count difference in the control samples between the two experiments. For annotation of post-translational modifications, we used the PhosphoSitePlus database.^59^

#### qPCR

72 h after base editing (induced by the addition of doxycycline), RNA was extracted and genomic DNA was removed (RNeasy columns and DNase I; Qiagen), followed by cDNA synthesis with SuperScript IV and random hexamers, and analysis using SYBR Green reagents on the Step One Plus (Thermo Fisher Scientific), with the following primers: Human *JAK1* 5′-GAGACAGGTCTCCCACAAACAC-3′, 5′-GTGGTAAGGACATCGCTTTTCCG-3′, Human *GAPDH* 5′-GTCTCCTCTGACTTCAACAGCG-3′, 5′-ACCACCCTGTTGCTGTAGCCAA-3′.

#### Giemsa staining

After six days of selection with IFNγ (1500 U/mL; Thermo Fisher Scientific), cells were washed with PBS, fixed with 4% PFA for 20 min and then stained with Giemsa working solution (1X in water; Sigma-Aldrich) for 2 h at room temperature with gentle rocking. Wells were rinsed with deionized water three times and then allowed to dry before images were taken by scanning.

### Quantification and statistical analysis

Statistical tests, exact value and description of n, definition of center, dispersion and precision measures are described in the figure legends. No randomization was performed and no statistical methods were used for sample size determination. For CRISPR-Cas9 screening analysis with MAGeCK, p < 0.05 and a false discovery of <5% were used as significance thresholds. For Student’s t-test, significance was defined as p < 0.05.

### Additional resources

BE-view is an R Shiny app that facilitates exploration of our data: www.sanger.ac.uk/tool/be-view.

## Data Availability

• All sequencing data have been released to the European Nucleotide Archive (ENA) for public access. Accessions can be found in Table S6. Read counts for CRISPR and base editing screens are available in Tables S1 and S2. • Data wrangling for graphs was performed with R and code can be found here: https://github.com/MatthewACoelho/Base_Editing_Screens. • Any additional information required to reanalyze the data reported in this paper is available from the lead contact upon request.

## Supplemental information

Document S1. Figures S1–S7

Table S1, CRISPR-Cas9 screens, Related to Figure 1

Table S2, BE screens, Related to Figure 2, Figure 3, Figure 4

Table S3, Base edited variants, Related to Figure 4

Table S4, Single-cell DNA-seq, Related to Figure 6

Table S5, Addgene deposit, Related to STAR Methods

Table S6, ENA accessions, Related to STAR Methods

Table S7, DNA-seq primers, Related to STAR Methods

Document S2. Article plus supplemental information

## References

[1] Lupardus P.J., Ultsch M., Wallweber H., Bir Kohli P., Johnson A.R., Eigenbrot C. (2014). Structure of the pseudokinase-kinase domains from protein kinase TYK2 reveals a mechanism for Janus kinase (JAK) autoinhibition. Proc. Natl. Acad. Sci. USA.

[2] Schwartz D.M., Kanno Y., Villarino A., Ward M., Gadina M., O'Shea J.J. (2017). JAK inhibition as a therapeutic strategy for immune and inflammatory diseases. Nat. Rev. Drug Discov..

[3] Kaplan D.H., Shankaran V., Dighe A.S., Stockert E., Aguet M., Old L.J., Schreiber R.D. (1998). Demonstration of an interferon gamma-dependent tumor surveillance system in immunocompetent mice. Proc. Natl. Acad. Sci. USA.

[4] Sharma P., Hu-Lieskovan S., Wargo J.A., Ribas A. (2017). Primary, adaptive, and acquired resistance to cancer immunotherapy. Cell.

[5] Zaretsky J.M., Garcia-Diaz A., Shin D.S., Escuin-Ordinas H., Hugo W., Hu-Lieskovan S., Torrejon D.Y., Abril-Rodriguez G., Sandoval S., Barthly L. (2016). Mutations associated with acquired resistance to PD-1 blockade in melanoma. N. Engl. J. Med..

[6] Shin D.S., Zaretsky J.M., Escuin-Ordinas H., Garcia-Diaz A., Hu-Lieskovan S., Kalbasi A., Grasso C.S., Hugo W., Sandoval S., Torrejon D.Y. (2017). Primary resistance to PD-1 blockade mediated by JAK1/2 mutations. Cancer Discov..

[7] Gao J., Shi L.Z., Zhao H., Chen J., Xiong L., He Q., Chen T., Roszik J., Bernatchez C., Woodman S.E. (2016). Loss of IFN-gamma pathway genes in tumor cells as a mechanism of resistance to anti-CTLA-4 therapy. Cell.

[8] Sucker A., Zhao F., Pieper N., Heeke C., Maltaner R., Stadtler N., Real B., Bielefeld N., Howe S., Weide B. (2017). Acquired IFNgamma resistance impairs anti-tumor immunity and gives rise to T-cell-resistant melanoma lesions. Nat. Commun..

[9] Rooney M.S., Shukla S.A., Wu C.J., Getz G., Hacohen N. (2015). Molecular and genetic properties of tumors associated with local immune cytolytic activity. Cell.

[10] Patel S.J., Sanjana N.E., Kishton R.J., Eidizadeh A., Vodnala S.K., Cam M., Gartner J.J., Jia L., Steinberg S.M., Yamamoto T.N. (2017). Identification of essential genes for cancer immunotherapy. Nature.

[11] Larson R.C., Kann M.C., Bailey S.R., Haradhvala N.J., Llopis P.M., Bouffard A.A., Scarfó I., Leick M.B., Grauwet K., Berger T.R. (2022). CAR T cell killing requires the IFNgammaR pathway in solid but not liquid tumours. Nature.

[12] Tate J.G., Bamford S., Jubb H.C., Sondka Z., Beare D.M., Bindal N., Boutselakis H., Cole C.G., Creatore C., Dawson E. (2019). COSMIC: the catalogue of somatic mutations in cancer. Nucleic Acids Res..

[13] Alexandrov L.B., Nik-Zainal S., Wedge D.C., Aparicio S.A.J.R., Behjati S., Biankin A.V., Bignell G.R., Bolli N., Borg A., Børresen-Dale A.L. (2013). Signatures of mutational processes in human cancer. Nature.

[14] Kweon J., Jang A.H., Shin H.R., See J.E., Lee W., Lee J.W., Chang S., Kim K., Kim Y. (2020). A CRISPR-based base-editing screen for the functional assessment of BRCA1 variants. Oncogene.

[15] Després P.C., Dubé A.K., Seki M., Yachie N., Landry C.R. (2020). Perturbing proteomes at single residue resolution using base editing. Nat. Commun..

[16] Jun S., Lim H., Chun H., Lee J.H., Bang D. (2020). Single-cell analysis of a mutant library generated using CRISPR-guided deaminase in human melanoma cells. Commun. Biol..

[17] Cuella-Martin R., Hayward S.B., Fan X., Chen X., Huang J.W., Taglialatela A., Leuzzi G., Zhao J., Rabadan R., Lu C. (2021). Functional interrogation of DNA damage response variants with base editing screens. Cell.

[18] Hanna R.E., Hegde M., Fagre C.R., DeWeirdt P.C., Sangree A.K., Szegletes Z., Griffith A., Feeley M.N., Sanson K.R., Baidi Y. (2021). Massively parallel assessment of human variants with base editor screens. Cell.

[19] Sánchez-Rivera F.J., Diaz B.J., Kastenhuber E.R., Schmidt H., Katti A., Kennedy M., Tem V., Ho Y.J., Leibold J., Paffenholz S.V. (2022). Base editing sensor libraries for high-throughput engineering and functional analysis of cancer-associated single nucleotide variants. Nat. Biotechnol..

[20] Kim Y., Lee S., Cho S., Park J., Chae D., Park T., Minna J.D., Kim H.H. (2022). High-throughput functional evaluation of human cancer-associated mutations using base editors. Nat. Biotechnol..

[21] Komor A.C., Kim Y.B., Packer M.S., Zuris J.A., Liu D.R. (2016). Programmable editing of a target base in genomic DNA without double-stranded DNA cleavage. Nature.

[22] Gaudelli N.M., Komor A.C., Rees H.A., Packer M.S., Badran A.H., Bryson D.I., Liu D.R. (2017). Programmable base editing of A^∗^T to G^∗^C in genomic DNA without DNA cleavage. Nature.

[23] Anzalone A.V., Koblan L.W., Liu D.R. (2020). Genome editing with CRISPR-Cas nucleases, base editors, transposases and prime editors. Nat. Biotechnol..

[24] Coelho M.A., de Carné Trécesson S., Rana S., Zecchin D., Moore C., Molina-Arcas M., East P., Spencer-Dene B., Nye E., Barnouin K. (2017). Oncogenic RAS signaling promotes tumor immunoresistance by stabilizing PD-L1 mRNA. Immunity.

[25] Gstalder C., Liu D., Miao D., Lutterbach B., DeVine A.L., Lin C., Shettigar M., Pancholi P., Buchbinder E.I., Carter S.L. (2020). Inactivation of Fbxw7 impairs dsRNA sensing and confers resistance to PD-1 blockade. Cancer Discov..

[26] Bock C., Datlinger P., Chardon F., Coelho M.A., Dong M.B., Lawson K.A., Lu T., Maroc L., Norman T.M., Song B. (2022). High-content CRISPR screening. Nat. Rev. Methods Primers.

[27] Shifrut E., Carnevale J., Tobin V., Roth T.L., Woo J.M., Bui C.T., Li P.J., Diolaiti M.E., Ashworth A., Marson A. (2018). Genome-wide CRISPR screens in primary human T cells reveal key regulators of immune function. Cell.

[28] Vredevoogd D.W., Kuilman T., Ligtenberg M.A., Boshuizen J., Stecker K.E., de Bruijn B., Krijgsman O., Huang X., Kenski J.C.N., Lacroix R. (2019). Augmenting immunotherapy impact by lowering tumor TNF cytotoxicity threshold. Cell.

[29] Lawson K.A., Sousa C.M., Zhang X., Kim E., Akthar R., Caumanns J.J., Yao Y., Mikolajewicz N., Ross C., Brown K.R. (2020). Functional genomic landscape of cancer-intrinsic evasion of killing by T cells. Nature.

[30] Manguso R.T., Pope H.W., Zimmer M.D., Brown F.D., Yates K.B., Miller B.C., Collins N.B., Bi K., LaFleur M.W., Juneja V.R. (2017). In vivo CRISPR screening identifies Ptpn2 as a cancer immunotherapy target. Nature.

[31] Dong M.B., Wang G., Chow R.D., Ye L., Zhu L., Dai X., Park J.J., Kim H.R., Errami Y., Guzman C.D. (2019). Systematic immunotherapy target discovery using genome-scale in vivo CRISPR screens in CD8 T cells. Cell.

[32] Pan D., Kobayashi A., Jiang P., Ferrari de Andrade L., Tay R.E., Luoma A.M., Tsoucas D., Qiu X., Lim K., Rao P. (2018). A major chromatin regulator determines resistance of tumor cells to T cell-mediated killing. Science.

[33] Le D.T., Uram J.N., Wang H., Bartlett B.R., Kemberling H., Eyring A.D., Skora A.D., Luber B.S., Azad N.S., Laheru D. (2015). PD-1 blockade in tumors with mismatch-repair deficiency. N. Engl. J. Med..

[34] Behan F.M., Iorio F., Picco G., Gonçalves E., Beaver C.M., Migliardi G., Santos R., Rao Y., Sassi F., Pinnelli M. (2019). Prioritization of cancer therapeutic targets using CRISPR-Cas9 screens. Nature.

[35] Hart T., Chandrashekhar M., Aregger M., Steinhart Z., Brown K.R., MacLeod G., Mis M., Zimmermann M., Fradet-Turcotte A., Sun S. (2015). High-Resolution CRISPR screens reveal fitness genes and genotype-specific cancer liabilities. Cell.

[36] Hart T., Moffat J. (2016). BAGEL: a computational framework for identifying essential genes from pooled library screens. BMC Bioinf..

[37] Li W., Xu H., Xiao T., Cong L., Love M.I., Zhang F., Irizarry R.A., Liu J.S., Brown M., Liu X.S. (2014). MAGeCK enables robust identification of essential genes from genome-scale CRISPR/Cas9 knockout screens. Genome Biol..

[38] Colic M., Wang G., Zimmermann M., Mascall K., McLaughlin M., Bertolet L., Lenoir W.F., Moffat J., Angers S., Durocher D., Hart T. (2019). Identifying chemogenetic interactions from CRISPR screens with drugZ. Genome Med..

[39] Szklarczyk D., Gable A.L., Lyon D., Junge A., Wyder S., Huerta-Cepas J., Simonovic M., Doncheva N.T., Morris J.H., Bork P. (2019). STRING v11: protein-protein association networks with increased coverage, supporting functional discovery in genome-wide experimental datasets. Nucleic Acids Res..

[40] Kroczynska B., Rafidi R.L., Majchrzak-Kita B., Kosciuczuk E.M., Blyth G.T., Jemielity J., Warminska Z., Saleiro D., Mehrotra S., Arslan A.D. (2016). Interferon gamma (IFNgamma) signaling via mechanistic target of rapamycin complex 2 (mTORC2) and regulatory effects in the generation of type II interferon biological responses. J. Biol. Chem..

[41] van der Meer D., Barthorpe S., Yang W., Lightfoot H., Hall C., Gilbert J., Francies H.E., Garnett M.J. (2019). Cell Model Passports-a hub for clinical, genetic and functional datasets of preclinical cancer models. Nucleic Acids Res..

[42] Liau N.P.D., Laktyushin A., Lucet I.S., Murphy J.M., Yao S., Whitlock E., Callaghan K., Nicola N.A., Kershaw N.J., Babon J.J. (2018). The molecular basis of JAK/STAT inhibition by SOCS1. Nat. Commun..

[43] Apriamashvili G., Vredevoogd D.W., Krijgsman O., Bleijerveld O.B., Ligtenberg M.A., de Bruijn B., Boshuizen J., Traets J.J.H., D'Empaire Altimari D., van Vliet A. (2022). Ubiquitin ligase STUB1 destabilizes IFNgamma-receptor complex to suppress tumor IFNgamma signaling. Nat. Commun..

[44] Coelho M.A., Li S., Pane L.S., Firth M., Ciotta G., Wrigley J.D., Cuomo M.E., Maresca M., Taylor B.J.M. (2018). BE-FLARE: a fluorescent reporter of base editing activity reveals editing characteristics of APOBEC3A and APOBEC3B. BMC Biol..

[45] Doench J.G., Fusi N., Sullender M., Hegde M., Vaimberg E.W., Donovan K.F., Smith I., Tothova Z., Wilen C., Orchard R. (2016). Optimized sgRNA design to maximize activity and minimize off-target effects of CRISPR-Cas9. Nat. Biotechnol..

[46] Toubiana J., Okada S., Hiller J., Oleastro M., Lagos Gomez M., Aldave Becerra J.C., Ouachée-Chardin M., Fouyssac F., Girisha K.M., Etzioni A. (2016). Heterozygous STAT1 gain-of-function mutations underlie an unexpectedly broad clinical phenotype. Blood.

[47] Nishimasu H., Shi X., Ishiguro S., Gao L., Hirano S., Okazaki S., Noda T., Abudayyeh O.O., Gootenberg J.S., Mori H. (2018). Engineered CRISPR-Cas9 nuclease with expanded targeting space. Science.

[48] Koblan L.W., Doman J.L., Wilson C., Levy J.M., Tay T., Newby G.A., Maianti J.P., Raguram A., Liu D.R. (2018). Improving cytidine and adenine base editors by expression optimization and ancestral reconstruction. Nat. Biotechnol..

[49] Komor A.C., Zhao K.T., Packer M.S., Gaudelli N.M., Waterbury A.L., Koblan L.W., Kim Y.B., Badran A.H., Liu D.R. (2017). Improved base excision repair inhibition and bacteriophage Mu Gam protein yields C:G-to-T:A base editors with higher efficiency and product purity. Sci. Adv..

[50] Doman J.L., Raguram A., Newby G.A., Liu D.R. (2020). Evaluation and minimization of Cas9-independent off-target DNA editing by cytosine base editors. Nat. Biotechnol..

[51] Kim Y.B., Komor A.C., Levy J.M., Packer M.S., Zhao K.T., Liu D.R. (2017). Increasing the genome-targeting scope and precision of base editing with engineered Cas9-cytidine deaminase fusions. Nat. Biotechnol..

[52] Richter M.F., Zhao K.T., Eton E., Lapinaite A., Newby G.A., Thuronyi B.W., Wilson C., Koblan L.W., Zeng J., Bauer D.E. (2020). Phage-assisted evolution of an adenine base editor with improved Cas domain compatibility and activity. Nat. Biotechnol..

[53] Livesey B.J., Marsh J.A. (2020). Using deep mutational scanning to benchmark variant effect predictors and identify disease mutations. Mol. Syst. Biol..

[54] Landrum M.J., Lee J.M., Benson M., Brown G.R., Chao C., Chitipiralla S., Gu B., Hart J., Hoffman D., Jang W. (2018). ClinVar: improving access to variant interpretations and supporting evidence. Nucleic Acids Res..

[55] Miao D., Margolis C.A., Vokes N.I., Liu D., Taylor-Weiner A., Wankowicz S.M., Adeegbe D., Keliher D., Schilling B., Tracy A. (2018). Genomic correlates of response to immune checkpoint blockade in microsatellite-stable solid tumors. Nat. Genet..

[56] Van Allen E.M., Miao D., Schilling B., Shukla S.A., Blank C., Zimmer L., Sucker A., Hillen U., Foppen M.H.G., Goldinger S.M. (2015). Genomic correlates of response to CTLA-4 blockade in metastatic melanoma. Science.

[57] Rizvi N.A., Hellmann M.D., Snyder A., Kvistborg P., Makarov V., Havel J.J., Lee W., Yuan J., Wong P., Ho T.S. (2015). Cancer immunology. Mutational landscape determines sensitivity to PD-1 blockade in non-small cell lung cancer. Science.

[58] Roh W., Chen P.L., Reuben A., Spencer C.N., Prieto P.A., Miller J.P., Gopalakrishnan V., Wang F., Cooper Z.A., Reddy S.M. (2017). Integrated molecular analysis of tumor biopsies on sequential CTLA-4 and PD-1 blockade reveals markers of response and resistance. Sci. Transl. Med..

[59] Snyder A., Makarov V., Merghoub T., Yuan J., Zaretsky J.M., Desrichard A., Walsh L.A., Postow M.A., Wong P., Ho T.S. (2014). Genetic basis for clinical response to CTLA-4 blockade in melanoma. N. Engl. J. Med..

[60] Riaz N., Havel J.J., Makarov V., Desrichard A., Urba W.J., Sims J.S., Hodi F.S., Martín-Algarra S., Mandal R., Sharfman W.H. (2017). Tumor and microenvironment evolution during immunotherapy with nivolumab. Cell.

[61] Karczewski K.J., Francioli L.C., Tiao G., Cummings B.B., Alföldi J., Wang Q., Collins R.L., Laricchia K.M., Ganna A., Birnbaum D.P. (2020). The mutational constraint spectrum quantified from variation in 141, 456 humans. Nature.

[62] Cattaneo C.M., Dijkstra K.K., Fanchi L.F., Kelderman S., Kaing S., van Rooij N., van den Brink S., Schumacher T.N., Voest E.E. (2020). Tumor organoid-T-cell coculture systems. Nat. Protoc..

[63] Dijkstra K.K., Cattaneo C.M., Weeber F., Chalabi M., van de Haar J., Fanchi L.F., Slagter M., van der Velden D.L., Kaing S., Kelderman S. (2018). Generation of tumor-reactive T cells by Co-culture of peripheral Blood lymphocytes and tumor organoids. Cell.

[64] Chow R.D., Michaels T., Bellone S., Hartwich T.M., Bonazzoli E., Iwasaki A., Song E., Santin A.D. (2022). Distinct mechanisms of mismatch repair deficiency delineate two modes of response to PD-1 immunotherapy in endometrial carcinoma. Cancer Discov..

[65] Litchfield K., Reading J.L., Puttick C., Thakkar K., Abbosh C., Bentham R., Watkins T.B.K., Rosenthal R., Biswas D., Rowan A. (2021). Meta-analysis of tumor- and T cell-intrinsic mechanisms of sensitization to checkpoint inhibition. Cell.

[66] Jenkins R.W., Aref A.R., Lizotte P.H., Ivanova E., Stinson S., Zhou C.W., Bowden M., Deng J., Liu H., Miao D. (2018). Ex vivo profiling of PD-1 blockade using organotypic tumor spheroids. Cancer Discov..

[67] Skoulidis F., Goldberg M.E., Greenawalt D.M., Hellmann M.D., Awad M.M., Gainor J.F., Schrock A.B., Hartmaier R.J., Trabucco S.E., Gay L. (2018). STK11/LKB1 mutations and PD-1 inhibitor resistance in KRAS-mutant lung adenocarcinoma. Cancer Discov..

[68] Shalem O., Sanjana N.E., Hartenian E., Shi X., Scott D.A., Mikkelson T., Heckl D., Ebert B.L., Root D.E., Doench J.G., Zhang F. (2014). Genome-scale CRISPR-Cas9 knockout screening in human cells. Science.

[69] Hugo W., Zaretsky J.M., Sun L., Song C., Moreno B.H., Hu-Lieskovan S., Berent-Maoz B., Pang J., Chmielowski B., Cherry G. (2016). Genomic and transcriptomic features of response to anti-PD-1 therapy in metastatic melanoma. Cell.

[70] Schmiedel J.M., Lehner B. (2019). Determining protein structures using deep mutagenesis. Nat. Genet..

[71] Findlay G.M., Daza R.M., Martin B., Zhang M.D., Leith A.P., Gasperini M., Janizek J.D., Huang X., Starita L.M., Shendure J. (2018). Accurate classification of BRCA1 variants with saturation genome editing. Nature.

[72] Muiños F., Martínez-Jiménez F., Pich O., Gonzalez-Perez A., Lopez-Bigas N. (2021). In silico saturation mutagenesis of cancer genes. Nature.

[73] Anzalone A.V., Randolph P.B., Davis J.R., Sousa A.A., Koblan L.W., Levy J.M., Chen P.J., Wilson C., Newby G.A., Raguram A., Liu D.R. (2019). Search-and-replace genome editing without double-strand breaks or donor DNA. Nature.

[74] Tian R., Gachechiladze M.A., Ludwig C.H., Laurie M.T., Hong J.Y., Nathaniel D., Prabhu A.V., Fernandopulle M.S., Patel R., Abshari M. (2019). CRISPR interference-based platform for multimodal genetic screens in human iPSC-derived neurons. Neuron.

[75] Fu J., Li Q., Liu X., Tu T., Lv X., Yin X., Lv J., Song Z., Qu J., Zhang J. (2021). Human cell based directed evolution of adenine base editors with improved efficiency. Nat. Commun..

[76] Hodgkins A., Farne A., Perera S., Grego T., Parry-Smith D.J., Skarnes W.C., Iyer V. (2015). WGE: a CRISPR database for genome engineering. Bioinformatics.

[77] Billon P., Bryant E.E., Joseph S.A., Nambiar T.S., Hayward S.B., Rothstein R., Ciccia A. (2017). CRISPR-mediated base editing enables efficient disruption of eukaryotic genes through induction of STOP codons. Mol. Cell.

[78] Koike-Yusa H., Li Y., Tan E.P., Velasco-Herrera M.D.C., Yusa K. (2014). Genome-wide recessive genetic screening in mammalian cells with a lentiviral CRISPR-guide RNA library. Nat. Biotechnol..

[79] Coelho M.A., De Braekeleer E., Firth M., Bista M., Lukasiak S., Cuomo M.E., Taylor B.J.M. (2020). CRISPR GUARD protects off-target sites from Cas9 nuclease activity using short guide RNAs. Nat. Commun..

[80] Zhu S., Cao Z., Liu Z., He Y., Wang Y., Yuan P., Li W., Tian F., Bao Y., Wei W. (2019). Guide RNAs with embedded barcodes boost CRISPR-pooled screens. Genome Biol..

[81] Jones D., Raine K.M., Davies H., Tarpey P.S., Butler A.P., Teague J.W., Nik-Zainal S., Campbell P.J. (2016). cgpCaVEManWrapper: simple execution of CaVEMan in order to detect somatic single nucleotide variants in NGS data. Curr. Protoc. Bioinformatics.

[82] Danecek P., Bonfield J.K., Liddle J., Marshall J., Ohan V., Pollard M.O., Whitwham A., Keane T., McCarthy S.A., Davies R.M., Li H. (2021). Twelve years of SAMtools and BCFtools. GigaScience.

[83] Yates L.R., Knappskog S., Wedge D., Farmery J.H.R., Gonzalez S., Martincorena I., Alexandrov L.B., Van Loo P., Haugland H.K., Lilleng P.K. (2017). Genomic evolution of breast cancer metastasis and relapse. Cancer Cell.

[84] McLaren W., Gil L., Hunt S.E., Riat H.S., Ritchie G.R.S., Thormann A., Flicek P., Cunningham F. (2016). The ensembl variant effect predictor. Genome Biol..

[85] Ran F.A., Hsu P.D., Wright J., Agarwala V., Scott D.A., Zhang F. (2013). Genome engineering using the CRISPR-Cas9 system. Nat. Protoc..

[86] Tzelepis K., Koike-Yusa H., De Braekeleer E., Li Y., Metzakopian E., Dovey O.M., Mupo A., Grinkevich V., Li M., Mazan M. (2016). A CRISPR dropout screen identifies genetic vulnerabilities and therapeutic targets in acute myeloid leukemia. Cell Rep..

