# Supplementary material for: Base editing screens map mutations affecting interferon-γ signaling in cancer
Source: Cancer Cell. 2023 Feb 13;41(2):288–303.e6. doi: 10.1016/j.ccell.2022.12.009 (PMC9942875; doi:10.1016/j.ccell.2022.12.009)
Supplement: Document S1. Figures S1–S7 [file mmc1.pdf]

Cancer Cell, Volume *41*

Supplemental information

Base editing screens map mutations affecting interferon-γ signaling in cancer

Matthew A. Coelho, Sarah Cooper, Magdalena E. Strauss, Emre Karakoc, Shriram Bhosle, Emanuel Gonçalves, Gabriele Picco, Thomas Burgold, Chiara M. Cattaneo, Vivien Veninga, Sarah Consonni, Cansu Dinçer, Sara F. Vieira, Freddy Gibson, Syd Barthorpe, Claire Hardy, Joel Rein, Mark Thomas, John Marioni, Emile E. Voest, Andrew Bassett, and Mathew J. Garnett

Supplemental Figure 1

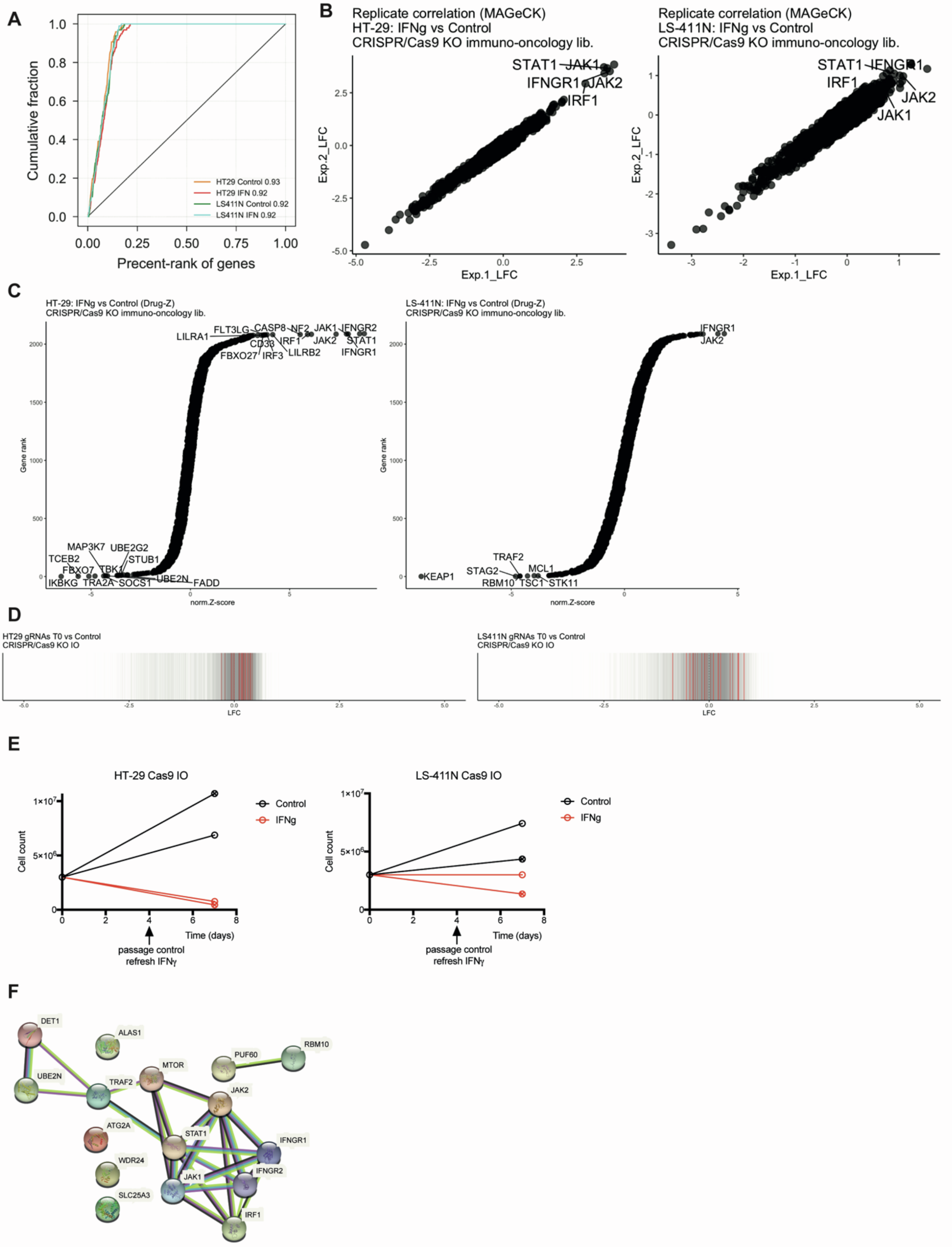

Figure S1. CRISPR-Cas9 screens identify mediators of IFNγ sensitivity and resistance, Related to Figure 1.

- **A)** Precision-recall analysis of CRISPR-Cas9 screen performance in HT-29, LS-411N cells with or without IFNγ. Precision-recall was based on the recovery of known essential genes versus the plasmid control, and the area under the curve is given in each case.
- **B)** Replicate correlation from MAGeCK analysis of CRISPR-Cas9 screens (control vs IFNγ arms) based on gRNA log2 fold-changes. Top resistance hits are shown for each cell line.
- **C)** Drug-Z analysis of averaged CRISPR-Cas9 screens (control vs IFNγ arms) with top hits indicated for each cell line.
- **D)** MAGeCK analysis of CRISPR-Cas9 screens (control vs T0 arms) showing individual gRNAs targeting *JAK1*, *JAK2*, *IFNGR1*, *IFNGR2*, *STAT1*, *IRF1*, in red.
- **E)** Growth curves showing cell proliferation in two independent CRISPR-Cas9 immuno-oncology target screens performed in HT-29 and LS-411N CRC Cas9-expressing cell lines. Arrow indicates when the cells were passaged in the control arm, whereas at this point in the IFNγ arm, IFNγ was refreshed.
- **F)** STRING network analysis of protein interactions for IFNγ-sensitizing and resistance genes common to HT-29 and LS-411N.

Supplemental Figure 2

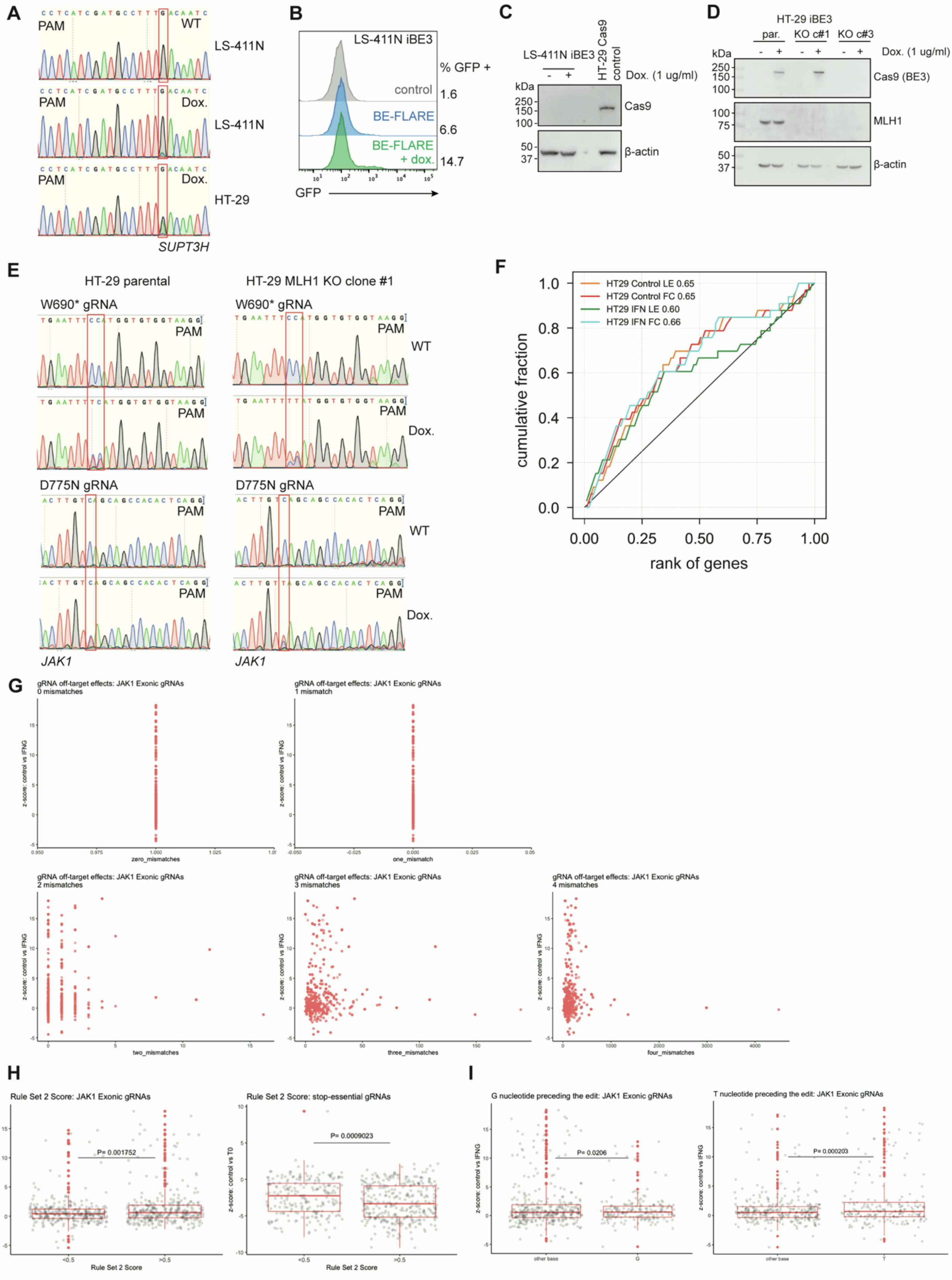

Figure S2. Base editing mutagenesis screening of *JAK1* variants, Related to Figure 2.

- **A)** Sanger sequencing analysis of the *SUPT3H* locus targeted with BE3 in HT-29 and LS-411N iBE3 cells. G->A editing is observed with the addition of doxycycline for 72 h. The protospacer sequence is displayed.
- **B)** BE-FLARE reporter assessment of base editing activity in LS-411N iBE3-NGG cells, 72 h after the addition of doxycycline, based on flow cytometry analysis of a BFP (His66) to GFP (Ty66) spectral shift. The percentage of cells that are GFP positive (base edited) are measured with flow cytometry. Data are representative of two independent experiments performed on separate days.
- **C)** Western blot analysis of LS-411N iBE3 cells 48 h after induction of base editor expression with doxycycline. Cas9 was not detected. HT-29 Cas9 serves as a positive control for Cas9 detection. Data are representative of two independent experiments performed on separate days.
- **D)** Western blot analysis of HT29 iBE3 *MLH1* KO single cell clone (KO c#3). KO was performed using transient expression of a CRISPR-Cas9 plasmid co-expressing a gRNA against *MLH1*.
- **E)** Sanger sequencing analysis of base editing of JAK1 loci using the indicated gRNAs in HT-29 iBE3 and HT-29 iBE3 *MLH1* KO cells. Base editing was induced with doxycycline for 72 h.
- **F)** Precision-recall analysis of base editing screen performance in HT-29 iBE3 cells in the control or IFNγ arms based on the recall of known essential genes. Area under the curve is given in each case for Drug-Z analysis of average control vs time zero (T0) conditions from two independent replicate screens. (FACS screen, fc; Proliferation screen, Le).
- **G)** Off-target analysis of JAK1 base editing library. Plotted are the proliferation screen z-scores (control vs IFNγ arms) against the number of off-target genomic positions (with 0 = on-target, 1, 2, 3 and four mismatches) for each gRNA targeting JAK1 exonic regions.
- **H)** gRNAs targeting JAK1 exons or generating stop codons in essential genes were assigned a Rule Set 2 Score and grouped into <0.5 or >0.5. Proliferation screen z-scores were compared between groups using an unpaired, two-tailed Student's t-test. Box and whiskers plot: center line, median; box limits, upper and lower quartiles; whiskers, 1.5× interquartile range; points, outliers.
- **I)** gRNAs targeting JAK1 exons were grouped by the predicted edited cytosine's direct genomic context; preceded by a G or preceded by a T. Proliferation screen z-scores were compared between groups using an unpaired, two-tailed Student's t-test. Box and whiskers plot: center line, median; box limits, upper and lower quartiles; whiskers, 1.5× interquartile range; points, outliers.

Supplemental Figure 3

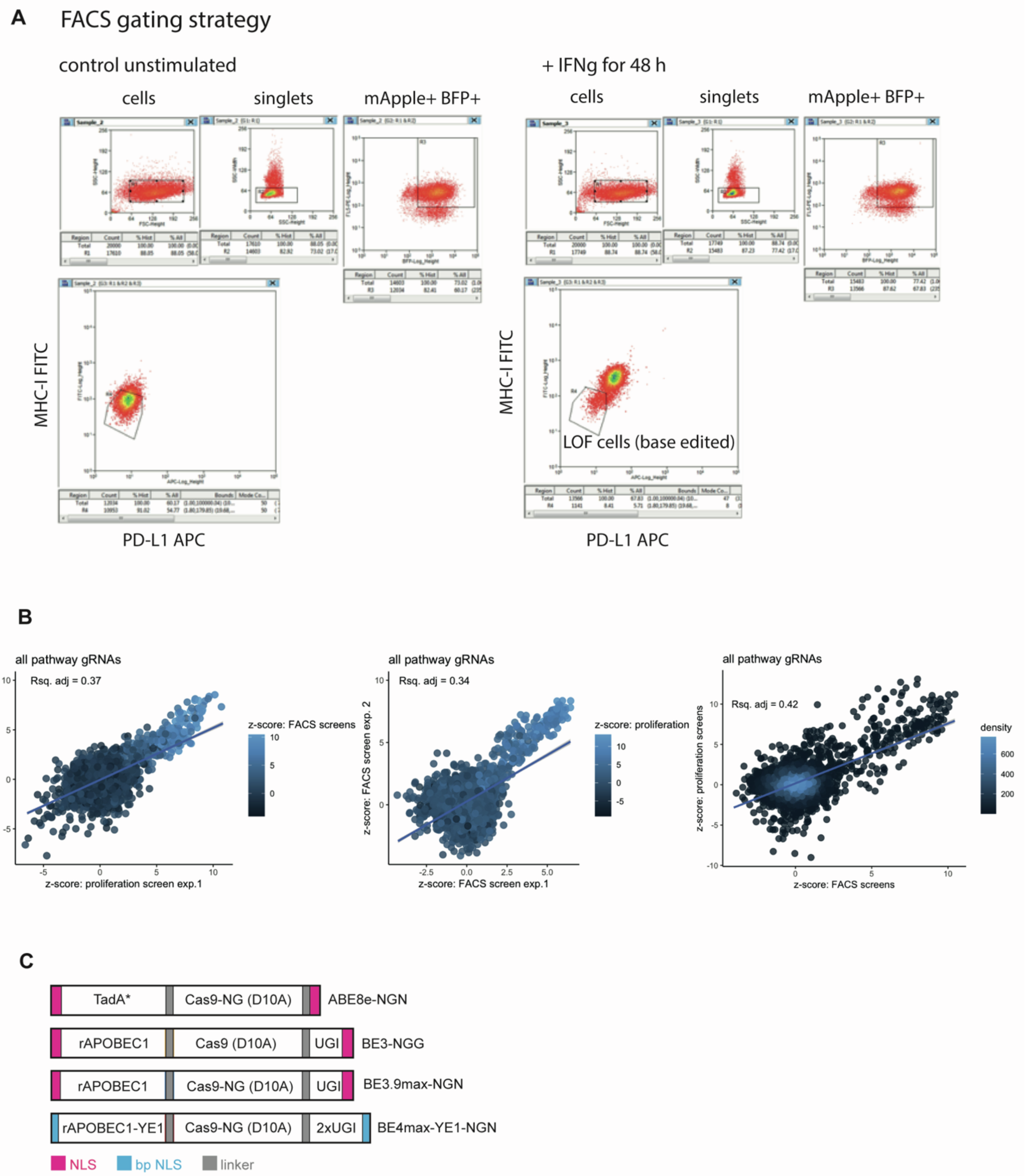

Figure S3. Base editing mutagenesis of the IFNγ pathway, Related to Figure 3 and Figure 4.

A) FACS gating strategy for cells with LOF in the IFNγ pathway. HT-29 iBE3 cells were stimulated with IFNγ (400 U/ml) for 48 h before FACS. Single cells expressing base editor (mApple) and gRNA (BFP) were gated and the cells unable to induce PD-L1 and MHC-I were gated based on an unstimulated control population. Data are representative of two independent experiments performed on separate days.

B) Replicate correlation for base editor screening of the IFNγ pathway. Correlation between z-scores for independent base editor screening replicate experiments performed on separate days, and independent screening assays (FACS and proliferation). Correlation between proliferation screens; R^2adj. 0.37; FACS screens; R^2adj. 0.34. Correlation between proliferation and FACS screens; R^2adj. 0.42.

C) Schematic of base editor architectures used in screening experiments. Bp NLS; bipartite nuclear localization sequence. TadA* denotes evolved TadA monomer.

Supplemental Figure 4

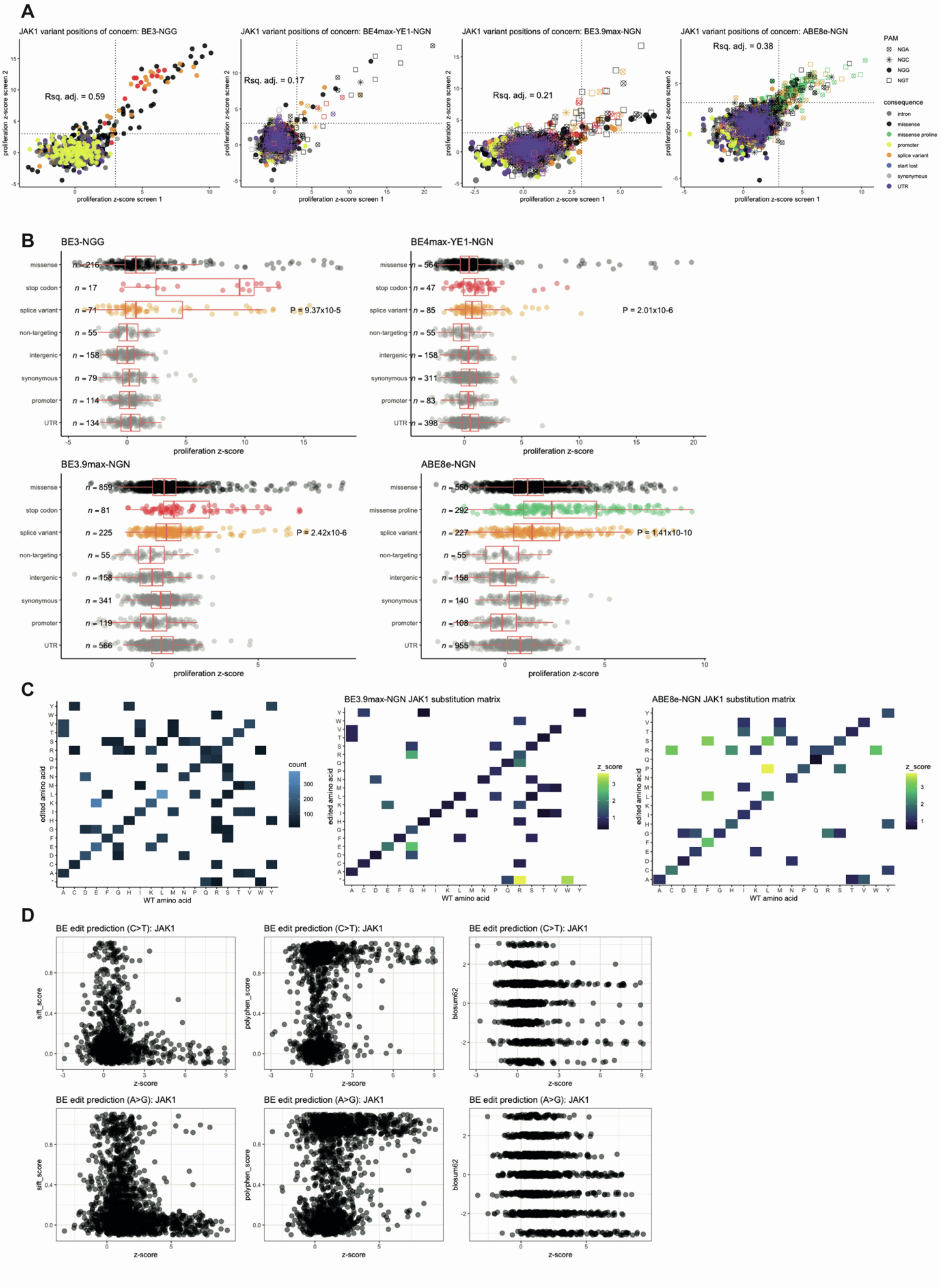

Figure S4. Base editing reveals JAK1 LOF and GOF variants with clinical precedence, Related to Figure 4.

- **A)** Replicate correlation of base editing screens using different base editor architectures and deaminases. Dot plots of gRNAs targeting JAK1 are colored by predicted consequence. Shape indicates PAM usage of the gRNA and adjusted R^2 values are indicated. z-scores (control vs IFNγ-arms; proliferation screens) are from two independent screens performed on separate days.
- **B)** Boxplot of proliferation screen z-scores for gRNAs by predicted consequence. Z-scores for predicted splice variant and non-targeting gRNAs (control vs IFNγ-arms) were compared using an unpaired, two-tailed Student's t-test. Shown is the median, box limits are upper and lower quartiles, whiskers are 1.5× interquartile range, and points are outliers.
- **C)** Heatmap showing the frequency of predicted amino acid substitutions in JAK1 when merging CBE and ABE-NGN base editing screens, and (right) aggregated predicted codon changes for each gRNA targeting JAK1 and gRNA z-scores from control vs IFNγ-arms for BE3.9max-NGN and ABE8e-NGN proliferation screens.
- **D)** Comparison of bioinformatic prediction of variant effect with experimental data from base editing screens (z-scores from control vs IFNγ-arms; proliferation screens). SIFT (0 is deleterious, 1 is tolerated), PolyPhen (0 is benign, 1 is damaging) and BLOSUM62 (positive is conserved, negative is not conserved).

Supplemental Figure 5

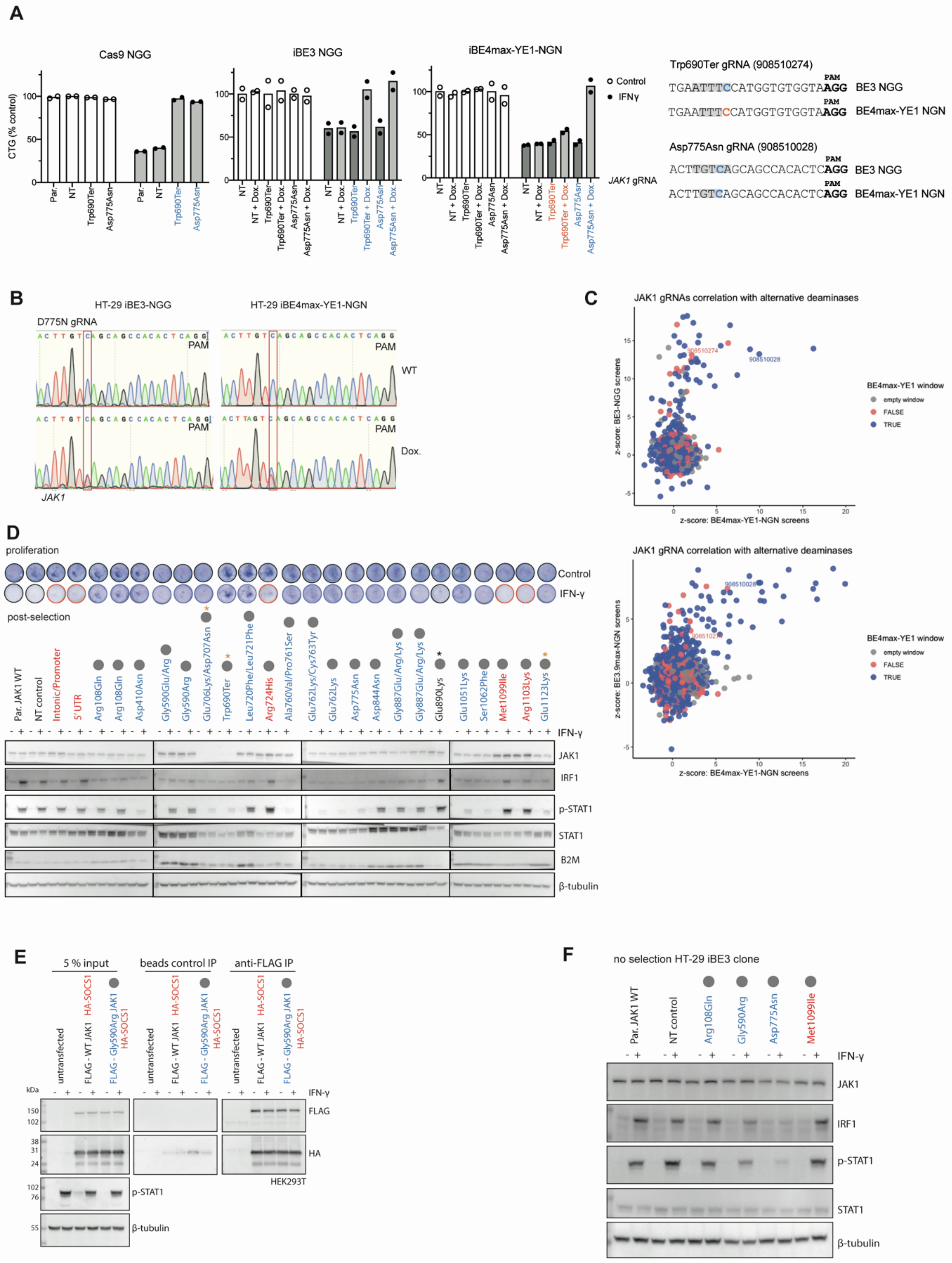

Figure S5. Functional validation of base editing variants conferring altered sensitivity to IFNγ, Related to Figure 5.

A) Comparison of gene editing technologies. Cas9-NGG or doxycycline-inducible BE3-NGG or BE4max-YE1-NGN were compared by measuring growth of HT-29 cells expressing the indicated gRNAs treated with IFNγ for 6 d. Data represent the mean of two independent experiments performed on separate days, with each experiment performed in technical triplicate. Two *JAK1* LOF gRNAs with targeted cytosines inside or outside of the predicted deaminase activity window (shaded grey).

B) Comparison of *JAK1* base editing efficiency by BE3-NGG and BE4max-YE1-NGN. Data for HT-29 iBE3 are also shown in Fig. S2.

C) Correlation between gRNA performance for gRNAs in both iBE3-NGG and iBE4max-YE1-NGN, and iBE3.9-NGN and iBE4max-NGN screens. gRNAs with a target cytosine within the narrower iBE4max-YE1-NGN deaminase activity window are shown in blue. gRNA IDs relating to other Figures are shown for reference.

D) Validation of JAK1 variants by Western blotting. Independent experiments replicating phenotypes described in Fig. 5B. Grey circles indicate variants with clinical precedence.

E) Immunoprecipitation analysis of HA-SOCS1 and FLAG-JAK1 or FLAG-JAK1Gly590Arg mutant from transiently transfected HEK293T cells, with and without IFNγ stimulation.

F) Western blotting analysis of JAK1 expression and JAK-STAT signaling of corresponding JAK1 variants was performed on a HT-29 iBE3 clonal cell line with high editing efficiency, stimulated with IFNγ for 1 h, with no prior selection with IFNγ.

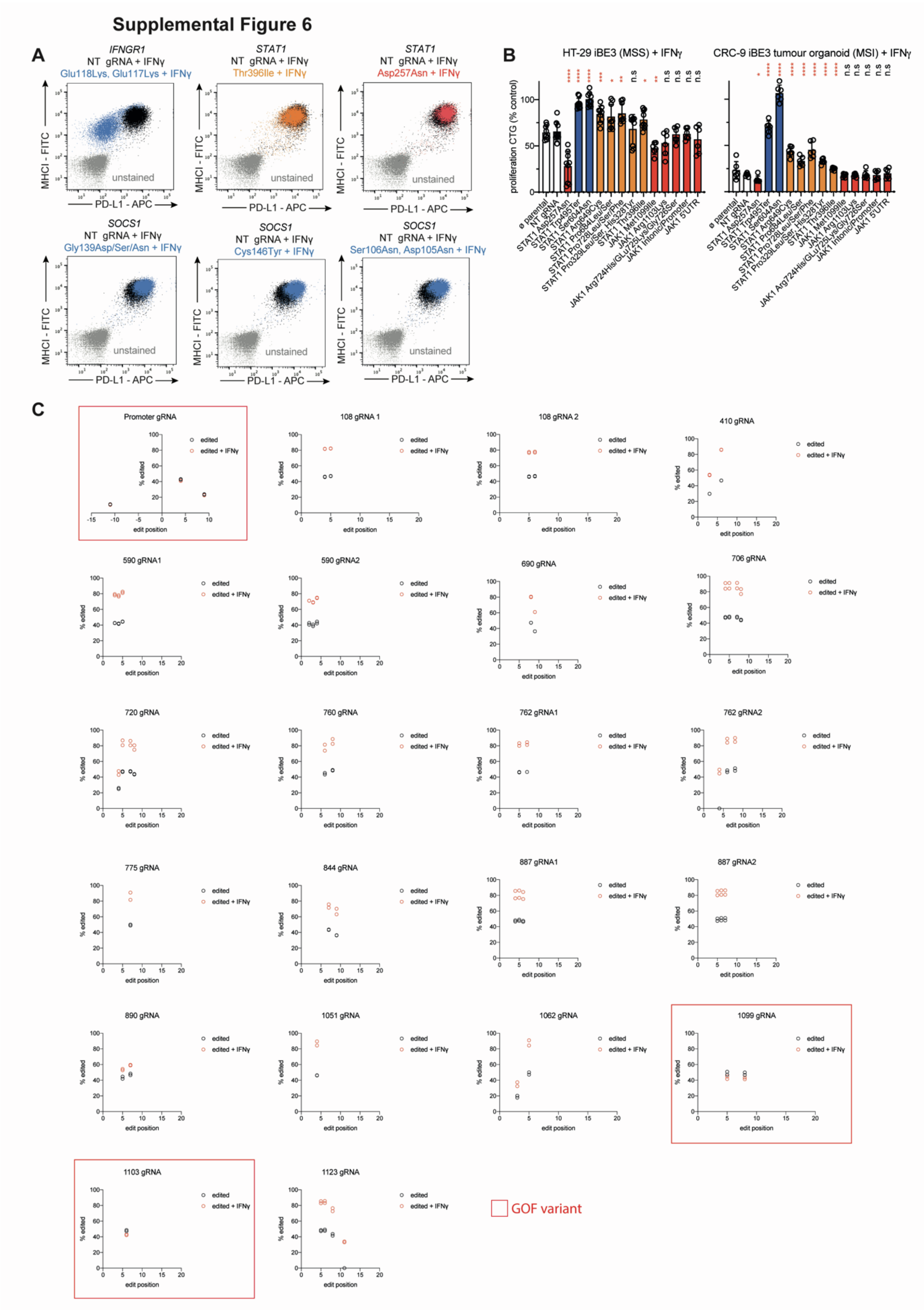

Supplemental Figure 6
A
IFNGR1
NT gRNA + IFNγ
Glu118Lys, Glu117Lys + IFNγ
STAT1
NT gRNA + IFNγ
Thr396Ile + IFNγ
STAT1
NT gRNA + IFNγ
Asp257Asn + IFNγ
SOCS1
NT gRNA + IFNγ
Gly139Asp/Ser/Asn + IFNγ
SOCS1
NT gRNA + IFNγ
Cys146Tyr + IFNγ
SOCS1
NT gRNA + IFNγ
Ser106Asn, Asp105Asn + IFNγ
MHCI - FITC
PD-L1 - APC
unstained
B
HT-29 iBE3 (MSS) + IFNγ
CRC-9 iBE3 tumour organoid (MSI) + IFNγ
proliferation CTG (% control)
n.s
C
Promoter gRNA
108 gRNA 1
108 gRNA 2
410 gRNA
590 gRNA1
590 gRNA2
690 gRNA
706 gRNA
720 gRNA
760 gRNA
762 gRNA1
762 gRNA2
775 gRNA
844 gRNA
887 gRNA1
887 gRNA2
890 gRNA
1051 gRNA
1062 gRNA
1099 gRNA
1103 gRNA
1123 gRNA
% edited
edit position
edited
edited + IFNγ
GOF variant

Figure S6. Amplicon sequencing of *JAK1* following base editing, Related to Figure 5 and Figure 6.

A) Representative flow cytometry plots relating to Figure 5C, showing induction of MHC-I and PD-L1 cell surface protein expression following IFNγ stimulation for 48 h in HT-29 iBE3 cells. The indicated base edited putative missense mutants show altered response; either increased expression (LOF in SOCS1), reduced expression (LOF in IFNGR1), or no change (separation of function, SOF, in STAT1). Data are representative of two independent experiments performed on separate days.

B) Cell Titer Glo cell proliferation assay comparing base edited mutant cancer cell lines, including STAT1 SOF mutants (orange) from Figure 5C. Data represent the mean $\pm$ SD of two (JAK1 putative GOF mutants and CRC-9 tumor organoid) or three (HT-29) independent experiments with three biological replicates per experiment. ****$P < 0.0001$, ***$P < 0.001$, **$P < 0.01$,*$P < 0.05$; n.s, not significant; unpaired, two-tailed Student's t-test compared to NT gRNA condition.

C) Amplicon sequencing of endogenous *JAK1* DNA reveals the editing profile of BE3 gRNAs. Position of edits relative to the protospacer are shown for LOF and GOF gRNAs in the validation cohort. Data are generated from control cells, cells with base editing or base editing and selection with IFNγ for 6 d. Data represent the mean of two independent experiments performed on separate days. Some of these data are also represented in Fig. 6B.

Supplemental Figure 7

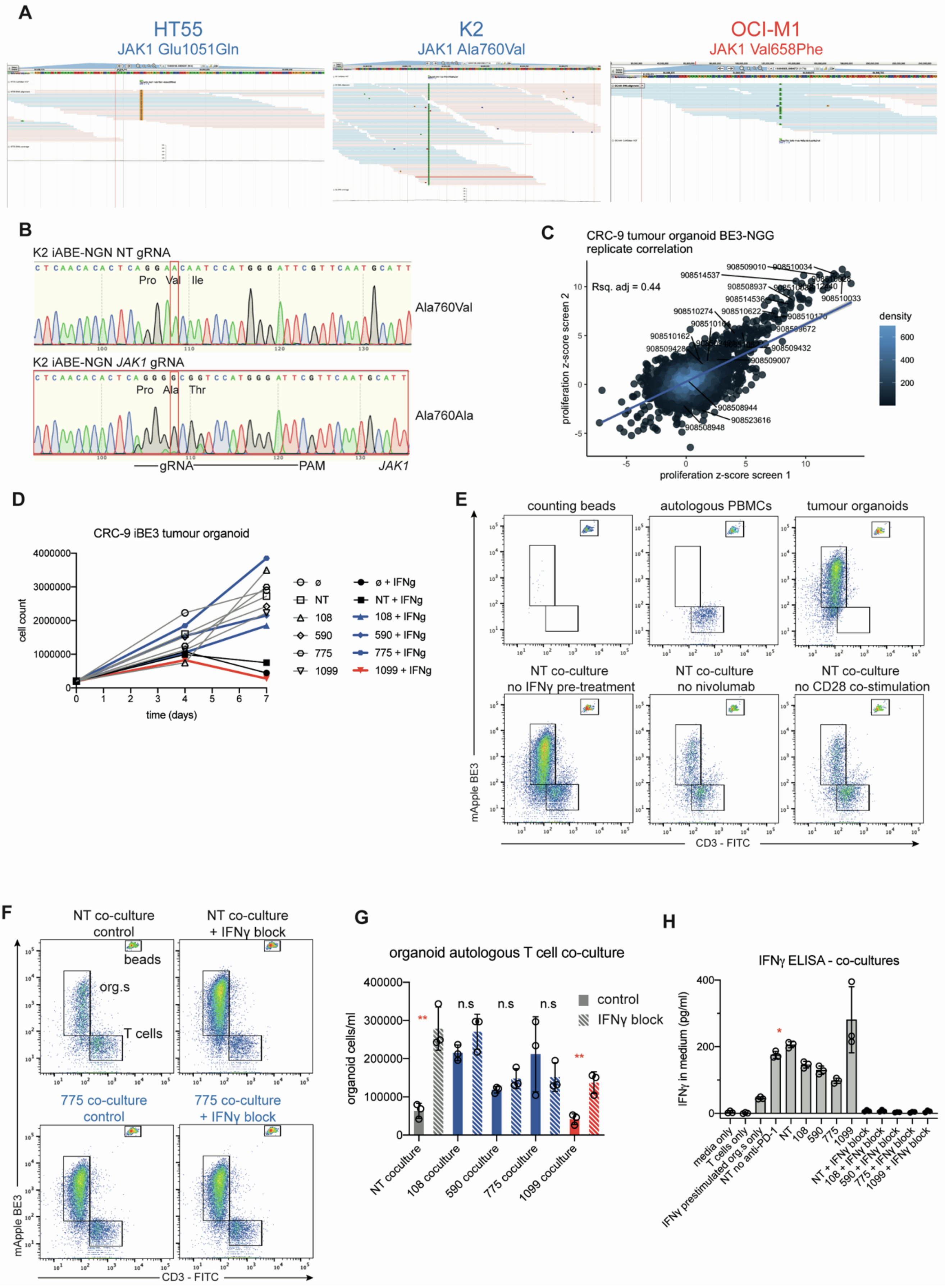

Figure S7. Classified *JAK1* missense mutations alter sensitivity to autologous anti-tumor T cells in primary human tumor organoids, Related to Figure 7.

- **A)** Exome sequencing data from HT55 and K2 cells lines with sequencing reads showing homozygous mutations in *JAK1*.
- **B)** MHC-I^{+} PD-L1^{+} cells from the experiment in Fig. 7B were sorted with FACS for DNA analysis by Sanger sequencing (right panel), revealing efficient reversion to WT JAK1 Ala760, and the bystander edit Ile759Thr. Data are representative of two independent experiments performed on separate days. NT; non-targeting control gRNA.
- **C)** Correlation between base editing replicate screens in CRC-9 tumor organoids. z-scores from the gRNAs targeting the IFNγ pathway were compared for independent replicate screens performed on separate days. gRNA IDs are labelled for the JAK1 validation cohort from iBE3-NGG JAK1 screens in the HT-29 cell model.
- **D)** Cell counts quantification of CRC-9 organoid growth in 3D, with (closed symbols) and without IFNγ (open symbols). JAK1 LOF mutants in blue grow progressively, whereas GOF JAK1 mutants in red, or controls in black, stop growing. Data are representative of two independent experiments performed on separate weeks.
- **E)** Representative flow cytometry plots and controls from T-cell and autologous tumor organoid co-cultures. Top panel shows counting beads, PBMCs or tumor organoids alone. Bottom panel show co-cultures after 3 d, where there is no organoids pre-treatment with IFNγ, no anti-PD-1 nivolumab in the co-culture, or no anti-CD28 co-stimulation. Data are representative of two-three biological replicates in each case.
- **F)** Representative flow cytometry plots from T-cell and autologous tumor organoid co-cultures, showing the protective effect of adding a neutralizing antibody against IFNγ in the medium. Data are representative of three biological replicates.
- **G)** Quantification of tumor organoid cell counts for autologous co-culture experiments with and without addition of a neutralizing antibody against IFNγ and different JAK1 LOF and GOF variants. Data represent the mean ± SD of three biological replicates. Comparison of IFNγ block and control conditions. $^{**}P < 0.01$; n.s, not significant; unpaired, two-tailed Student's t-test.
- **H)** ELISA measuring IFNγ release from anti-tumor T cells in PBMC co-culture with autologous tumor organoids. Data represent the mean ± SD of three biological replicates. Comparison of non-targeting (NT) co-culture with and without anti-PD1 nivolumab. $^{*}P < 0.05$ unpaired, two-tailed Student's t-test.
